# Developmental depression-to-facilitation shift controls excitation-inhibition balance

**DOI:** 10.1038/s42003-022-03801-2

**Published:** 2022-08-25

**Authors:** David W. Jia, Tim P. Vogels, Rui Ponte Costa

**Affiliations:** 1 Centre for Neural Circuits and Behaviour, Department of Physiology, Anatomy and Genetics, University of Oxford, Oxford, United Kingdom; 2 Institute of Science and Technology, Klosterneuburg, Austria; 3 Bristol Computational Neuroscience Unit, SCEEM, Faculty of Engineering, University of Bristol, Bristol, United Kingdom

**Keywords:** Synaptic development, Learning algorithms, Spike-timing-dependent plasticity, Neural circuits

## Abstract

Changes in the short-term dynamics of excitatory synapses over development have been observed throughout cortex, but their purpose and consequences remain unclear. Here, we propose that developmental changes in synaptic dynamics buffer the effect of slow inhibitory long-term plasticity, allowing for continuously stable neural activity. Using computational modeling we demonstrate that early in development excitatory short-term depression quickly stabilises neural activity, even in the face of strong, unbalanced excitation. We introduce a model of the commonly observed developmental shift from depression to facilitation and show that neural activity remains stable throughout development, while inhibitory synaptic plasticity slowly balances excitation, consistent with experimental observations. Our model predicts changes in the input responses from phasic to phasic-and-tonic and more precise spike timings. We also observe a gradual emergence of short-lasting memory traces governed by short-term plasticity development. We conclude that the developmental depression-to-facilitation shift may control excitation-inhibition balance throughout development with important functional consequences.

## Abstract (web summary)

Using computational modelling this study proposes that the commonly observed depression-to-facilitation shift across development controls excitation-inhibition balance in the brain.

## Introduction

Short-term synaptic plasticity is a hallmark of synaptic function. It refers to transient and fast changes in synaptic efficacy in the range of a few milliseconds up to several seconds^1–3^. Different short-term plasticity (STP) profiles regarding the direction and time-scale of change are found across cell types^4–7^, brain regions^8–12^, and throughout development^8–10,13–16^. For example, excitatory synapses from pyramidal cells in cortex are predominately short-term depressing in young animals, whereas adult synapses exhibit short-term facilitation (STF)^8^. Conversely, inhibitory synapses from cortical fast-spiking inhibitory interneurons are short-term depressing throughout development^4,6,7^. Functionally, STP is known to homeostatically control synaptic transmission and firing rates in neuronal networks on millisecond timescales^17–19^. However, it has remained unclear what is the combined impact of long-term and short-term synaptic plasticity for homeostatic control in neural circuits.

Recent studies suggest that long-term inhibitory plasticity (ISP)^20–24^, acting on the time-scale of minutes to hours, is also responsible for homeostasis, by way of establishing and maintaining excitation-inhibition balance, limiting the destabilizing effects of its excitatory counterpart^25,26^. However, the stabilizing effects of co-tuning excitatory and inhibitory synaptic currents, the hallmark of inhibitory synaptic plasticity, can only be observed in adult animals. In young animals, a tight excitation–inhibition balance has not yet formed and receptive fields are often unbalanced^26,27^. Despite this lack of detailed excitation–inhibition tuning, experimental observations consistently show that neural circuits exhibit stable firing activity at all stages of development^28–31^. Here, we hypothesize that STP provides the homeostatic control needed in young animals for low neural activity^32^.

Using computational models, we show how STP can complement and even control the expression of inhibitory long-term plasticity, thus acting as a gating mechanism for the emergence of excitation-inhibition balance across development. In particular, our model suggests that short-term depression (STD) is important to maintain stable neural activity even with flat inhibitory tuning curves in young animals^26^. Further, the gradual shift to STF, as observed throughout development^8–10,13,14,16^ allows for excitatory-inhibitory balance to emerge. We show that this developmental control of STP shapes neuronal dynamics, making neural responses more diverse and postsynaptic spike timings more precise over the course of maturation. Finally, the maturation of STP in our model leads to synapse-based short-lasting memory traces in an excitatory–inhibitory balanced model.

## Results

Changes in STP are a hallmark of neural development^8,12,33^, but their impact on neuronal dynamics has remained unclear. Here, we study the effects of STP in congruence with long-term ISP in a computational model of development, and show that STP can play a crucial role in young neurons, compensating for a lack of inhibitory tuning. Moreover, a gradual change of excitatory STP from depression to facilitation over development allows for excitatory-inhibitory balance to develop while guaranteeing stable response properties.

**Fig. 1:**
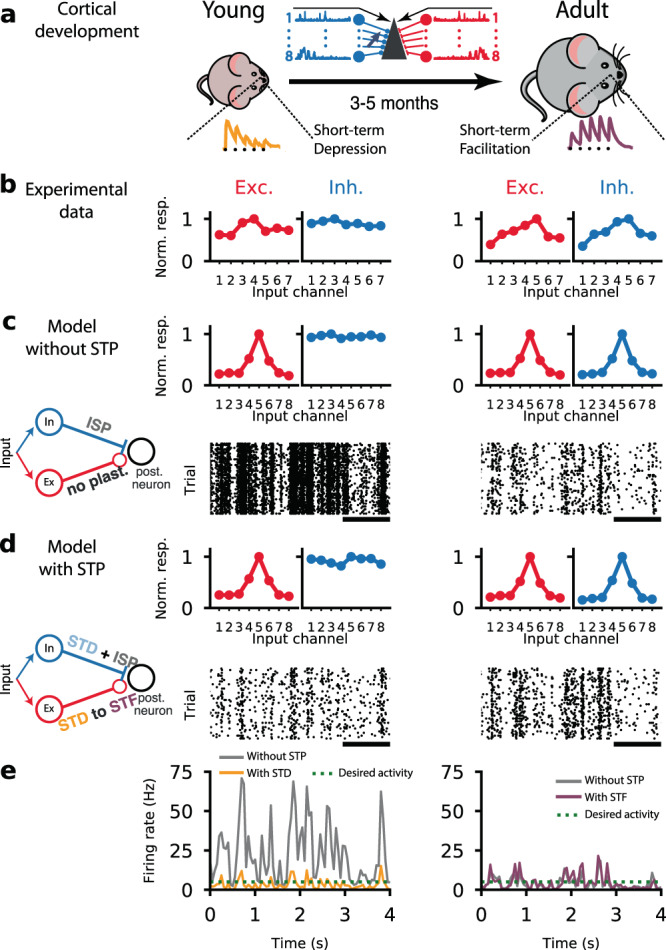
A feedfoward cortical circuit with short-term synaptic plasticity exhibits low firing rates in both young and adult conditions. **a** Schematic of animal development from young with short-term depression (left) to adult with short-term facilitation (right) at excitatory synapses as observed experimentally^8–10,13,14^. Traces of short-term synaptic plasticity (STP) for depression (orange) and facilitation (purple)^8^. In the middle is a schematic of the feedforward neural circuit with eight independent input channels, each with an excitatory (red) and an inhibitory (blue) group synapsing onto a postsynaptic neuron (Fig. S2). **b** Inhibitory tuning does not mirror excitatory tuning in young animals (left). Once animals reach adulthood, a precise excitation-inhibition (EI) balance can be observed. Panels adapted from a previous study^26^ . **c** Computational model with long-term synaptic plasticity in inhibitory synapses (ISP; see inset) started from unbalanced excitation-inhibition (top left) and gradually developed EI balance (top right). Neuron with unbalanced excitation-inhibition showed high activity (~20 Hz; bottom left), which was gradually reduced through ISP (~4.5 Hz; bottom right). Bottom raster plots represents postsynaptic spiking activity; black line corresponds to 1 second. **d** A computational model with both ISP and STP started from unbalanced excitation-inhibition (top left) and gradually developed EI balance (top right). Neuron with unbalanced excitation-inhibition shows low firing activity (~4.5 Hz; bottom left) throughout development (~4.5 Hz; bottom right). Bottom raster plots represents postsynaptic spiking activity; black line corresponds to 1 second. **e** Firing rates of a model without STP (left and right panels, solid gray line) and a model with both ISP and STP in young (left, solid orange line) and adult (right, solid purple line) conditions. Desired activity (dashed green line) represents baseline firing rate as observed experimentally^28–31^.

To investigate these effects, we built a model of a simple feedforward network with a single conductance-based integrate-and-fire neuron receiving inputs from 800 excitatory and 200 inhibitory afferents^22^. To emulate heterogeneous inputs we modeled eight different pathways (Fig. 1a) each with 100 excitatory and 25 inhibitory synapses, whose activity is determined by a time-varying rate signal (Methods). Excitatory and inhibitory synapses were modulated by STP, consistent with experimentally observed profiles in young and adult mice^8–10,12–14,33–37^. Inhibitory synapses additionally experienced long-term ISP^20,23^. Excitatory afferents were tuned according to experimentally observed receptive fields, while inhibitory baseline weights were initially flat (Fig. 1b, see also ref. ^26^).

Inhibitory long-term synaptic plasticity, working on a time-scale of hours, has been suggested to underlie excitation-inhibition (E-I) balance in cortical networks^20,22,23^. The slow nature of long-term synaptic plasticity is consistent with the gradual and slow development of E-I balance over multiple days from young to adult animals^26^ (Fig. 1b). However, the lack of detailed balance in young animals could lead to unstable, unnaturally high activity (Fig. 1c, e). This could, in principle, be compensated by increased learning rates at inhibitory synapses, but this is known to lead to unstable receptive field development^38,39^ and is not consistent with the slow and gradual development of E-I balance^25,26^.

STP can offer an elegant solution to maintain low firing rates throughout development. To this end, we added experimentally observed^4,6,7^ STD to all afferent synapses using a standard Tsodyks-Markram model^17^ (Methods). In contrast with the ISP-alone model, the addition of an appropriate STP profile that features STD at the excitatory synapses, led to lower firing rates in the “young” model, despite unbalanced excitation-inhibition (Fig. 1d, e).

Notably, the low postsynaptic firing rates that resulted from STD in the excitatory afferents effectively prevented long-term plasticity from developing inhibitory receptive fields that have been observed in adult animals (Fig. 1b;^26^). As we will see below, the shift of STP profiles over the course of development^8,12,33^ allowed the gradual tuning of inhibition in simulations of young-to-adult development.

To better highlight the respective points the results that follow are presented using different time courses. For ease of comparison all key results are also provided in Fig. S1 using the same time courses.

### Gradual depression-to-facilitation shift enables stable activity over development

Next we studied how the developmental changes of STD to STF in excitatory synapses^8–10,12–14,33–37^ may aid the tuning of inhibitory synapses by way of long-term plasticity, and provide stable postsynaptic firing rates throughout the process.

**Fig. 2:**
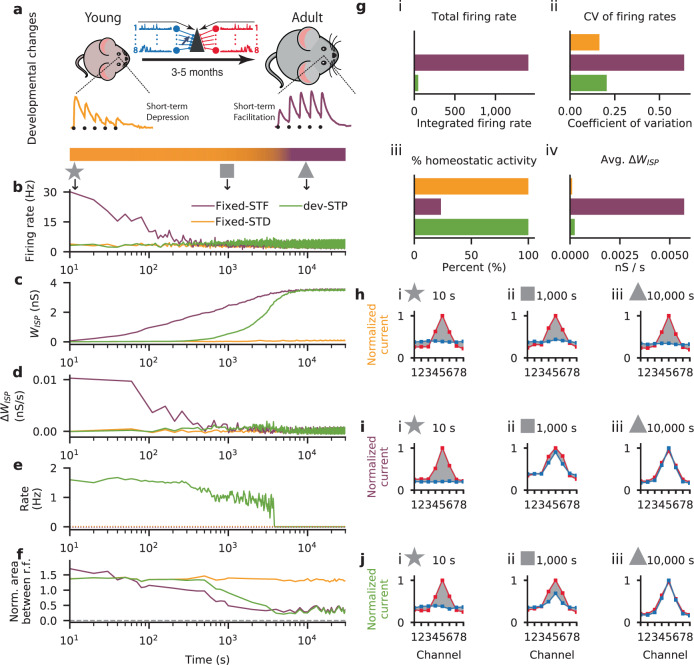
Gradual short-term plasticity shift maintained stable firing rates while detailed E-I balance developed. **a** Schematic of our developmental short-term plasticity (STP) model (cf. Fig. S2); top: young and adult STP (as in Fig. 1); bottom: gradual changes in STP from depressing to facilitating dynamics (orange and purple respectively, in log-scale as in **b**–**f**). **b**–**f** Different variables of the model across simulated development for three different models: fixed short-term depression (fixed-STD, orange), fixed short-term facilitation (fixed-STF, purple) and developmental model with gradual changes in STP (dev-STP, green line). Note *x*-axis on log-scale. **b** Receiver neuron firing rate. **c** Mean inhibitory weight. **d** Mean changes in the weight of the inhibitory synaptic afferents. **e** Rate of STP change (note that the curves for both fixed-STF and fixed-STD remain fixed at 0 as these models do not consider any developmental changes to STP, shown as dashed lines). **f** Area between normalized excitatory and inhibitory tuning curves (cf. **h**–**j**) during the course of simulated development. A normalized area close to 0 represents a perfectly balanced neuron. **g** Additional statistics for the three models. (i) Total neuronal activity calculated using the area between the firing rate in **b** and the desired target rate of 5 Hz. (ii) Average coefficient of variation of the firing rates across simulated development (cf. **b**). (iii) Percent of time spent under homeostasis (i.e., at the desired firing rate; cf. **b**). (iv) Average change in inhibitory weights (cf. **d**). **h**–**j** Snapshots of excitatory and inhibitory tuning curves across three points in simulated development: 10 s (star), 1000 s (square) and 10,000 s (triangle). Shaded gray area represents difference between excitatory and inhibitory tuning curves (cf. **f**). **h**–**j** Excitatory (red) and inhibitory (blue) postsynaptic tuning curves for the fixed-STD (**h**), fixed-STF (**i**) and dev-STP models (**j**).

To simulate ageing in our model, we devised an algorithm that gradually changed the STP parameters between young and adult profiles fitted to experimental data (Fig. 2a; Methods). The algorithm monitored average postsynaptic firing rate over sliding windows of 500 ms. When rates were stable and low, excitatory STP parameters were modified by a small amount towards facilitation (see Methods and Figs. S2–S4 for details on how the parameters and STP profiles were determined). For computational reasons we used a total simulation time of 8 hours to model development, but the exact temporal frame does not qualitatively change our results.

As expected the developmental STP model (dev-STP) maintained a low level of firing activity throughout the simulation (i.e., ~5 Hz in line with experimental observations in freely behaving rodents^32^) while a tight excitation-inhibition balance in the feedforward circuit developed (Fig. 2b). For clarity we use firing rates and synaptic input currents to assess the level of E-I balance, but the results are qualitative similar when considering other forms of measuring E–I balance (Fig. S5)^24,40^. As controls, we considered two other models in which STP was fixed either at STD (fixed-STD) or STF (fixed-STF) throughout the simulation. The fixed-STF scenario exhibited high and more variable firing rates before ISP was able to balance the postsynaptic neuron and lower the firing rates (Fig. 2b, g; Fig. S6). On the other hand, the fixed-STD scenario was able to maintain homeostatic balance throughout the simulation (Fig. 2b, g), but did not develop a tightly balanced inhibitory receptive field (Fig. 2f, h). In addition, to highlight the contribution of the different decisions made during model development we tested a number of model variations (see details in the Methods; Figs. S9–S11 and S13 for a summary plot).

Although the dev-STP and fixed-STF models converged to the same mean inhibitory weights (Fig. 2c), the fixed-STF scenario led to substantially higher firing rate variability during development, and large, somewhat erratic weight changes (Fig. 2g, d). In contrast dev-STP maintained relatively small weight changes throughout development (Fig. 2d). Finally, while the initial changes of receptive field in the fixed-STF scenario arose quickly, the time of convergence was similar to the dev-STP model (Fig. 2f, i, j), because long-term ISP in the dev-STP scenario sped up dramatically as facilitation developed (Fig. 2b–f). In the dev-STP model, ISP evolved the inhibitory tuning to match excitation (Fig. 2f), incrementally handing over control of the target firing rate to inhibition, which ensured postsynaptic activity remained relatively low (Fig. 2b). This means that each increase in the excitatory efficacy through strengthened STF was matched by an increase in the inhibitory efficacy through ISP, until inhibition was fully tuned and the excitatory synapses reach their adult profile of STF. Note that if ISP is not included in our model, the developmental changes of STD-to-STF at excitatory synapses would be prone to pathologically high firing rates^18,41^. Taken together, our results suggest the need for a synergistic interaction between excitatory short-term and inhibitory long-term synaptic plasticity.

**Fig. 3:**
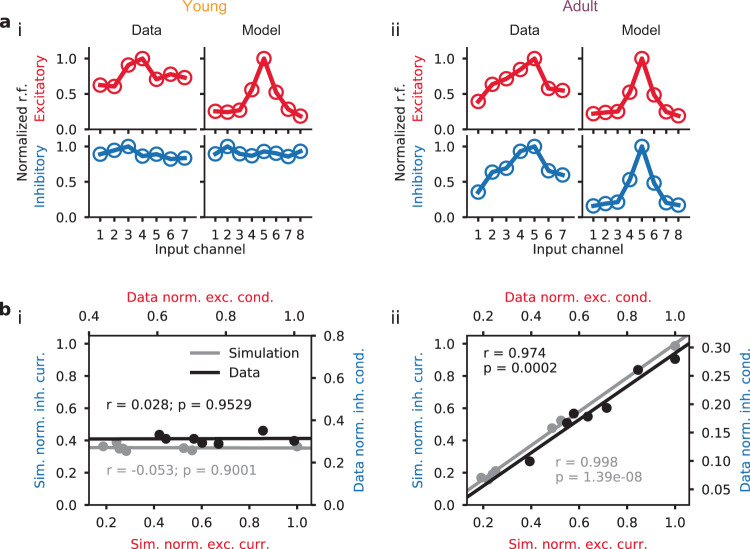
Depression-to-facilitation shift captured inhibitory receptive field development. **a** Comparison of experimentally observed and simulated (dev-STP model) excitatory and inhibitory tuning curves, for both young (i) and adult (ii) conditions. **b** Excitatory-inhibitory responses for model (gray) and experiments (black). Different dots represent different tone frequencies in the data and different input channels in the model. Lines represent linear correlation between excitatory and inhibitory responses in both model (gray) and experiments (black). Experimental data reproduced from a previous study^26^.

The dev-STP model was able to maintain the neuron in a (globally) balanced state throughout development while allowing inhibition to gradually mirror the excitatory tuning. In line with experimental in vivo observations in rat auditory cortex across development^26^ inhibitory tuning curves were initially flat (Fig. 3a). In the adult neuron, both model and experiment showed E-I balance. Using linear correlation analysis as done experimentally by^26^, we confirmed that excitatory and inhibitory responses in “young” models were not correlated, but became strongly correlated in the adult profile (Fig. 3b).

### Developmental changes in STP shape signal dynamics and transmission

**Fig. 4:**
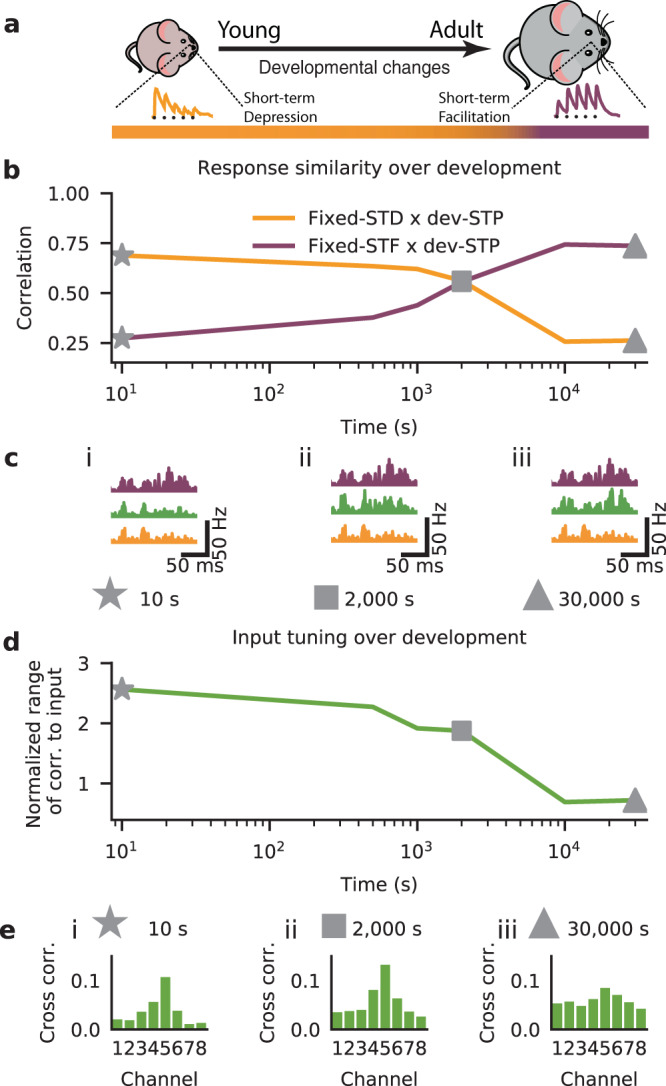
Input–output response correlations over development. **a** Schematic of the modeled development from young with depressing synapses (left) to adult facilitating synapses (right). Bottom color bar indicates the gradual shift in STP (as in Fig. 2). **b** Correlation of the dev-STP model response profiles to that of the fixed-STD (orange) and fixed-STF (purple) scenarios during development. **c** Example output responses (cf. Fig. S6) for the fixed-STD (orange), fixed-STF (purple), and dev-STP (green) models at three points in simulated development (i: 10 s, stars; ii: 2000 s, squares; iii: 30000 s, triangles). **d** Normalized range of correlation to input (Methods). **e** Example of output correlations at specific times during the course simulated development (same timings as in **c**). Results shown here were averaged over 50 trials.

Next we studied how the developmental STP model shapes neuronal responses and signal transmission. In line with the establishment of detailed balance^22^, the postsynaptic firing rates in the dev-STP model were initially more correlated with the fixed-STD model, and gradually became more correlated with the fixed-STF model (Fig. 4a–c; Fig. S6). Across all input channels we found a gradual decrease of input-output correlation (Fig. 4d). This was largely due to the fact that the output responses became less correlated with the preferred channel versus the non-preferred channels (Fig. 4e).

**Fig. 5:**
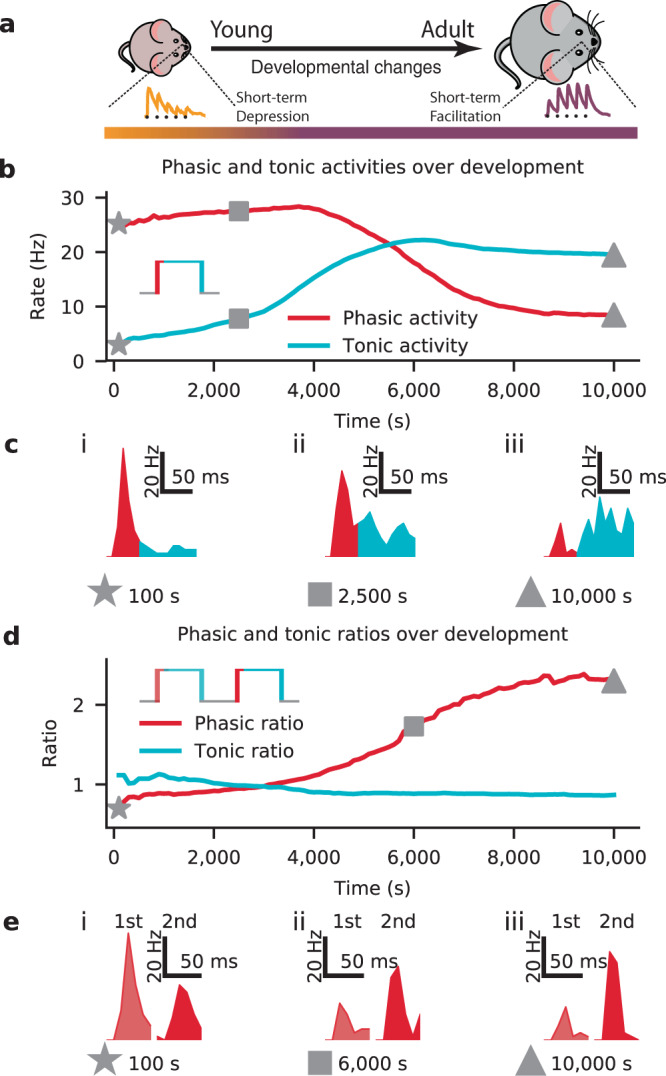
Developmental STP shaped tonic and phasic input-output transmission. **a** Schematic of the modeled development from young with depressing synapses (left) to adult facilitating synapses (right), as in previous figures. **b** Average phasic (red) and tonic (blue) postsynaptic firing rates for a step-input of 150 Hz (inset; cf. Fig. S6; see Fig. S1 for a comparison between these and other devSTP results). **c** Example output responses for the phasic (red) and tonic (blue) activities at three points during development. **d** Ratios of the average phasic (red) and tonic (blue) firing rates between two consecutive step stimuli (inset; see Fig. S1 for a comparison between these and other devSTP results). **e** Examples of responses to the first (light red) and second (dark red) phasic activities in response to the double step-input stimulus at specific points during development. Results shown here were averaged over 50 trials.

Another functional consequence of the changes in short-term dynamics could be observed in the phasic and tonic stimulus response profiles. Transient (phasic) and steady-state (tonic) neural activity has been observed in sensory cortical circuits^28,35,42,43^. We examined these properties by probing the neuron responses using a step-input stimulus (see Methods; Fig. 5b) to the preferred input channel (channel 5), simulating the sudden presence of a strong sensory feature. We defined the phasic response as the average activity over the first 50 ms after stimulus onset, and the tonic response as the average rate over the remaining stimulus duration (200 ms). Over development, the average phasic activity of the circuit decreased, while the tonic activity increased (Fig. 5b). These changes in the dynamics are a direct consequence of the gradual change from depressing to facilitating synapses, interacting with the strengthening inhibition (see Fig. S1 for comparison of results across figures). The shift in tonic and phasic responses to a single stimulus also affected subsequent input responses when using two paired step inputs (Fig. 5d inset; Methods). This interaction between subsequent responses was largest for the phasic response, which grew substantially over development, as seen by the increasing ratio of firing rate between the two stimuli (Fig. 5d, e). On the other hand, the tonic response decreased, but only slightly.

**Fig. 6:**
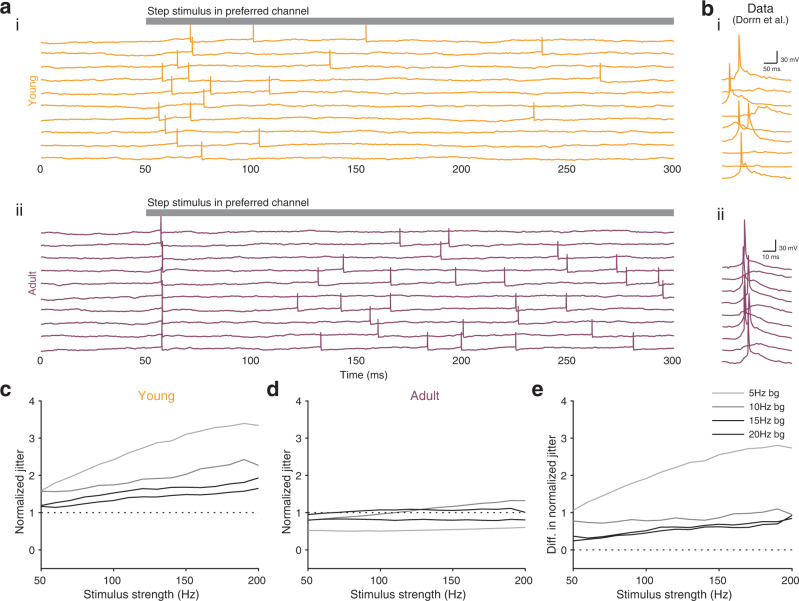
Adult STP improves temporal precision of postsynaptic spikes. **a** Examples of postsynaptic voltage responses with preferred channel input for both young STP model (i) and adult STP model (ii); gray bar at top represents time during which preferred channel is stimulated. **b** Stimulus evoked responses in in vivo recordings across a few trials in young (i) and adult (ii) animals. Panels adapted from a previous study^26^. In **a**, **b** the background firing rate is 5 Hz. **c**, **d** Normalized jitter of postsynaptic spikes in the young (**c**) and adult (**d**) model for different background firing rates (denoted by different shades of gray; see Methods; cf. Fig. S12 and Fig. S16). **e** Difference between normalized jitter of young STP model (**c**) and adult STP model (**d**).

We also investigated the phasic response to a step stimulus on very short time scales (Fig. 6a), specifically focusing on the temporal jitter of the first evoked spike (Fig. 6b). In line with experimental observations of reduced jitter over development^26^, we observed substantially more stimulus-locked spike times in the adult model than in the young model (Fig. 6c, d; Fig. S12). The young scenario showed higher normalized jitter (Methods) than the adult scenario across all stimulus strength, and particularly when the background activity before stimulus onset was low (Fig. 6e). This is the result of, under adult conditions, some of the connections do not resulting in any output spiking due to the tight excitation-inhibition balance. Finally, our results do not change qualitatively if non-normalized jitter is measured (Fig. S14).

### Emergence of short-lasting memory traces in a balanced neuron

Finally, we also investigated the longer term effects of changing STP over development with regard to its implications for short-lasting memory traces. STP has recently been proposed as a substrate for short-lasting memory traces^44,45^, owing to the fact that STF can promote increased response to previously displayed stimuli. Here, inspired by these ideas we used the dev-STP model to test for short-term memory properties in a balanced neuron. We compared the responses of a "recall" stimuli that were or were not preceded by a "preloaded" stimulus.

**Fig. 7:**
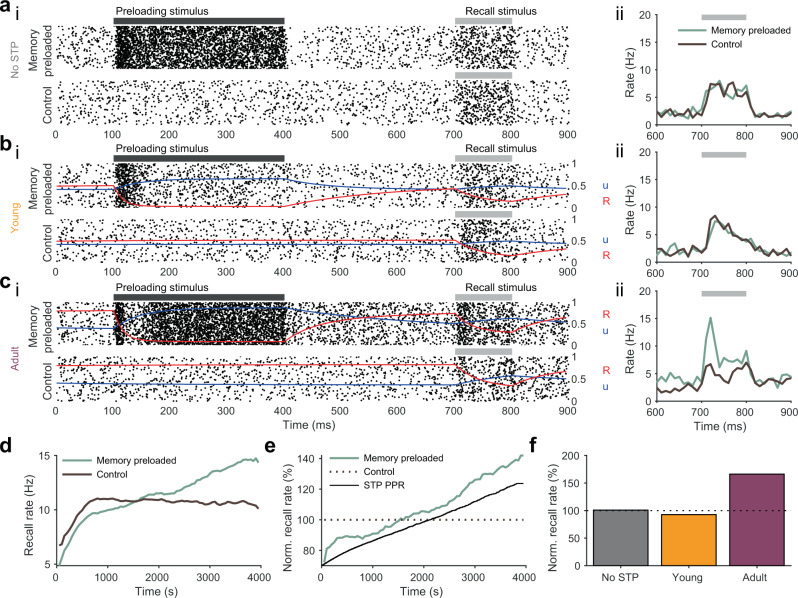
Gradual emergence of synaptic-based short-term memory traces over development. **a**–**c** Raster plot of short-term memory test (SMT, i-top)) with a preloaded stimulus and subsequent recall stimulus (black and gray bars, respectively) compared with raster plot of trials without the preloaded stimulus (i-bottom). Average firing rates (ii) for both memory preloaded (light green) and control conditions (dark brown). Release probability (u in blue) and number of presynaptic resources (R in red) are also given for reference (see Methods). **a** SMT in a model with only inhibitory synaptic plasticity (i.e. no STP). **b** SMT in a model with young STP profile. **c** SMT in a model with adult STP profile. **d** Firing rates during the recall period with (light green) or without (dark brown) preloaded stimulus. SMTs were preformed every 50 seconds during dev-STP development simulation (cf. Fig. 2) as STP changes from depression to facilitation at excitatory synapses. We only highlight the first 4000 s of the simulation as after this point changes in STP become minimal. **e** Normalized recall firing rates to the average firing rate of the control case (i.e., without memory preloading). The STP paired-pulse ratio (black) measuring the STP strength of the excitatory synapses for this period is also plotted as reference. **f** Normalized recall rate for three model conditions: no STP (gray), young STP (orange), and adult STP (purple).

Models with no STP mechanism, as well as the “young” dev-STP model showed identical firing rates during the recall period (Fig. 7a, b) independently of whether they had experienced a preloaded stimulus or not. This is because the form of STP that has been proposed to underpin (silent) short-term memory traces, that is STF, had not yet developed. The “adult” dev-STP model, on the other hand, showed substantially higher firing rates during the recall period (Fig. 7c, d) when the recall stimulus was preceded by a preloaded cue that activated STF in excitatory synapses. Dev-STP thus allowed the neuron to gradually utilize this silent short-term memory mechanism in a neuron with E-I balance (Figs. 3a, b and 7e, f). Note that this result depends on the dynamics of STF. For example, when adult facilitating synapses are replaced by mixed depression-facilitation, it results in much weaker memory traces (Fig. S15). This dependency on the STP profile suggests that short-lasting memories are more likely to be relevant to brain regions in which strong facilitating synapses are prevalent^44,45^.

## Discussion

It has been widely observed that short-term synaptic dynamics in the cortex change from depressing to facilitating throughout the course of development^8–10,12–14,33–37^. Here, we propose that this commonly observed shift in STP interacts with long-term plasticity at inhibitory synapses to form the fundamental architecture of neuronal processing. According to our model, short-term depressing synapses could help to stabilize neural networks in the absence of properly tuned inhibition in young animals (Figs. 1 and S6). A gradual change from STD to facilitation then allows for stable dynamics throughout development while inhibitory synaptic plasticity-mediated, detailed excitation-inhibition balance can emerge (Fig. 2). In addition to this stabilizing interplay, we show that the developmental maturation of STP also shapes signal processing, by allowing for more temporally precise coding (Fig. 6), and the emergence of synaptic-based short-lasting memory traces (Fig. 7).

There are currently two dominant views on how changes in STP throughout development may arise. One view is that these changes are caused by sensory experience^34^; the other view poses that these are hard-wired, pre-programmed changes^13^. Our developmental STP model suggests a way to reconcile these two views, in that both the sensory-dependent^34^ and non-sensory-dependent^13^ changes observed experimentally may be simply caused by changes in the neural baseline activity. However, although we have modeled changes in STP as a function of neural activity, it is in principle possible to allow for these changes to be purely hard-wired and continuous (cf. Fig. S11). In our hands, the latter mode, i.e. unilateral maturation of STP without heeding the co-development of inhibitory tuning curves, can also lead to stable development (Fig. S11), but this requires fine tuning of a STP change interval. Taken together with experimental observations^34^ these results suggest that activity-dependent changes provide a more biologically plausible mechanism for developmental STP, but further experimental work remains to be done to test these scenarios. In addition, our model also predicts that if this activity-dependent mechanism controlling dev-STP would be perturbed this would lead to a delayed onset of excitation-inhibition balance in cortical networks.

Concurrently with changes in STP there are also changes in baseline excitatory synaptic weights over development, which can increase, decrease or not change depending on experimental conditions^16,26^. We tested a range of variants of the dev-STP model, in which the baseline excitatory weight increases, decreases or does not change (Fig. S3v). Our results show that the qualitative outcome of dev-STP does not depend on how the baseline synaptic weight changes at excitatory synapses, suggesting that these modifications are not critical for the dev-STP functions studied here.

Our work highlights how developmental STP may shape temporal aspects of synaptic transmission. In particular, our model predicts that young animals primarily encode stimuli with transient, phasic activity, whereas adult animals may transmit both phasic transients and sustained tonic rates equally well. Interestingly, both modes of transmission have been observed in sensory cortices^28^ at different developmental stages. In our model we have assumed that STP changes at all excitatory synapses happen in lockstep over development. However, in the brain not all synapses are modified coincidentally^8–10^, and it is possible that this degree of variability gives a tighter homeostatic control throughout development.

We have focused on long-term inhibitory synaptic plasticity, but excitatory synapses also undergo long-term synaptic plasticity. Importantly, long-term excitatory synaptic plasticity also changes the short-term synaptic dynamics^19,46–48^. It is possible that the gradual changes of STP at excitatory synapses that we have considered here are mediated by long-term excitatory plasticity. Indeed, long-term modifications of presynaptic STP is mediated by retrograde signaling, which depends on postsynaptic activity, in line with our model^47–49^. In future work it would be interesting to explore the effects of long-term excitatory plasticity with realistic inputs in conjunction with inhibitory synaptic plasticity as a potential model for developmental STP^20,38,50^. In addition to the homeostatic mechanisms studied here (ISP and STP) there are others that we could have considered. However, the vast majority of the homeostatic mechanisms identified to date are relatively slow, so they would suffer from the same problem as ISP on its own – i.e. inability to quickly stabilize firing rates^51^. Therefore our model would also be relevant if other (slow) homeostatic mechanisms were included, but this remains to be tested in future work.

Our model shows a gradual increase in temporal precision of spiking over development, consistent with experimental observations in the auditory cortex of rats^26^, suggesting that STP maturation plays an important role in temporal encoding^52–56^. Our findings add to the growing experimental literature showing that inhibition-excitation balance sharpens spike timings^26,55,57,58^.

Here we have focused on a simple feedforward network. However, developmental changes in STP have also been found at recurrent synapses^8–10,13–16^. It has been shown previously that STD is important for a fast control of firing rates in recurrent synapses^59^. In addition due to strong instabilities common in recurrent networks, ISP is also critical in these cases^24^. This means that our combined model of dev-STP and ISP would be even more detrimental to avoid runaway and pathological firing rates (Fig. 1) when applied to a recurrent network. Recurrent neural network dynamics are traditionally thought of as being a property of working memory in the prefrontal cortex. However, STF at recurrent connections in the prefrontal cortex has been proposed as an alternative biologically plausible mechanism of working memory at the synaptic level^44,60,61^. Our short-term memory results (Fig. 7) resemble the working memory-like properties that have been proposed^44^. In future work it would be of interest to investigate how these translate into recurrent networks, which may offer a model with both E-I balance and synaptic-based working memory properties. This suggests that synaptic-based working memory properties may be more prevalent in adult cortices, enabling animals to retain information about the recent past throughout the brain.

We have demonstrated that the developmental shift towards STF may provide neural networks with the ability to encode short-term memory traces. However, there are other possible interpretations. In sensory systems plasticity processes across multi-timescales are known to facilitate sensory reactivation^62^, which may be relevant to the short-latency stimulus facilitation that we highlight above. In addition, it has been suggested recently that STD and facilitation play a critical role in hierarchical synaptic credit assignment across sensory and non-sensory streams^63,64^. The shift in STP that we study here may also contribute to this type of credit assignment throughout development.

Finally, dysfunctions in the regulation of excitation-inhibition balance underlie numerous neurological disorders^65–74^. In our model we show that STP can dynamically control the expression of long-term inhibitory synaptic plasticity, thus modulating E-I balance. Maldaptive developmental STP should thus be reflected in E-I malfunction. Interestingly, this is supported by disease animal models, in which STP and excitation-inhibition balance are both altered in animal models of dysplasia^75,76^.

Overall, our results suggest important functional roles for the commonly observed shift in STP during development.

## Materials and methods

### Neuron models

In this study, we used a conductance-based integrate-and-fire neuron model for simulations^77^. In this model, the membrane voltages are calculated following

1 $$\tau \frac{dV}{dt}=-{g}_{{{{{{{{\rm{leak}}}}}}}}}\cdot ({V}_{{{{{{{{\rm{rest}}}}}}}}}-V)+{g}_{{{{{{{{\rm{exc}}}}}}}}}\cdot ({E}_{{{{{{{{\rm{exc}}}}}}}}}-V)+{g}_{{{{{{{{\rm{inh}}}}}}}}}\cdot ({E}_{{{{{{{{\rm{inh}}}}}}}}}-V)$$

where *V* is the membrane potential of the neuron as a function of time *t*, *τ* is the membrane time constant, *V*_rest_ is the resting membrane potential, *E*_exc_ is the excitatory reversal potential, and *E*_inh_ is the inhibitory reversal potential. Our neuron parameters are the same as in previous studies^77^. In particular, we used a membrane capacitance, *C*, of 200pF with membrane resistance, *R*, of 100MΩ, which gives a membrane time constant *τ* = 20ms. *g*_exc_ and *g*_inh_, expressed in the units of the resting membrane conductance, are the synaptic conductances, and *g*_*l*_ is the leaky conductance. The synaptic conductances are modeled as ${\tau }_{{{{{{{{\rm{exc}}}}}}}}}\frac{d{g}_{{{{{{{{\rm{exc}}}}}}}}}}{dt}=-{g}_{{{{{{{{\rm{ex}}}}}}}}}$ and ${\tau }_{{{{{{{{\rm{inh}}}}}}}}}\frac{d{g}_{{{{{{{{\rm{inh}}}}}}}}}}{dt}=-{g}_{{{{{{{{\rm{in}}}}}}}}}$ where *τ*_exc_ and *τ*_inh_ are the synaptic time constants for the excitatory and the inhibitory conductances, respectively. When the neuron receives a presynaptic action potential, its conductance increases by *g*_exc_ → *g*_exc_ + *w*_exc_ or *g*_inh_ → *g*_inh_ + *w*_inh_ for excitatory and inhibitory synapses, respectively. The model parameters used are summarized in Table 1.

**Table 1:** Parameter values for conductance-based leaky integrate-and-fire model.

Parameter	Value
*τ*	20.0 ms
*R*	100.0 MΩ
*C*	200.0 pF
*g*_leak_	10.0 nS
*τ*_exc_	5.0 ms
*τ*_inh_	10.0 ms
*E*_exc_	0 mV
*E*_inh_	−70 mV
*V*_rest_	−60 mV
*V*_thresh_	−50 mV
*V*_reset_	−60 mV
*τ*_refrac_	4 ms

### Synaptic plasticity models

We used both STP and long-term inhibitory synaptic plasticity models in our work. Both were calculated separately in the simulations and combined as explained below.

#### Short-term synaptic plasticity

Short-term plasticity was used in the simulations following the Tsodyks-Markram (TM) model defined by^5,78,79^ as follows

2 $$\frac{dR(t)}{dt} 	=\frac{1-R(t)}{D}-u(t)R(t)\cdot \delta (t-{t}_{{{{{{{{\rm{AP}}}}}}}}})\\ \frac{du(t)}{dt} 	=\frac{U-u(t)}{F}+f\cdot (1-u(t))\cdot \delta (t-{t}_{{{{{{{{\rm{AP}}}}}}}}})$$

where *R* models vesicle depletion and *u* models the presynaptic release probability. Every presynaptic spike at *t*_AP_ causes a decrease in *R* by the number of vesicles available by *uR*, which then recovers exponentially to its baseline value of 1 with a time constant *D*. At the same time every presynaptic spike at *t*_AP_ also causes an increase in the release probability *u* by *f* ⋅ (1 − *u*(*t*)) (where *f* is the facilitation rate) and recovers exponentially to its baseline *U* with a time constant *F*. Finally, the postsynaptic potential, or the weight of the STP component for a synapse exhibiting STP at time *t* is computed as

3 $$w(t)=AR(t)u(t)$$

where *A* is the baseline amplitude factor. We use different weights *w*(*t*) for excitatory (*w*_exc_) and inhibitory (*w*_inh_) connections (see below for more details). In simulations, the initial value of *u* is set to *U*, and the initial value of *R* is set to 1. We used the four-parameter version of the TM model (*D*, *F*, *U*, *f*) as it provides an overall better fit of short-term dynamics data^79^.

#### STP model fitting

We found STP parameters which produced excitatory STP paired-pulse responses (PPRs) that matched those found in experiments for young and adult animals. Specifically, we used the STP PPRs observed experimentally^8^, with excitatory STP PPRs of 0.7 and 1.24 for young and adult animals respectively. In order to find STP parameter values that matched these PPRs, we interpolated between strong STD and strong STF parameter values^79^ (Fig. S2e). Using this interpolation we then calculated the PPR across all parameter sets. We use these PPRs to compare with experimental data from young and adult animals^8^. Finally we used least squares to obtain STP parameters that best matched the data in both young (STD) and adult conditions (STF) (see Table 2). For STP dynamics at inhibitory synapses, these are known to be short-term depressing (e.g.^80^) so we used the young case which follows short-term depressing dynamics (see Table 2).

**Table 2:** STP parameter values.

Synaptic dynamics	Connection	*D* (s)	*F* (s)	*U*	*f*	PPR
Depression	exc. (young); inh.	0.3134	0.0798	0.3917	0.062	0.70
Facilitation	exc. (adult)	0.0845	0.2959	0.1973	0.1168	1.24

Paired-pulse ratio (PPR) is given by dividing the second postsynaptic response by the first.

#### Inhibitory synaptic plasticity

Long-term ISP is implemented in all inhibitory synapses in all simulations unless otherwise specified. We used the same model as in previous studies^22^. In this model, each synapse *i* has a presynaptic trace *x*_*i*_, which increases with each spike by *x*_*i*_ → *x*_*i*_ + 1 and decays exponentially following ${\tau }_{{{{{{{{\rm{STDP}}}}}}}}}\frac{d{x}_{i}}{dt}=-{x}_{i}$. Then, the synaptic weight of a given synapse following pre- or postsynaptic spikes are updated by

4 $${A}_{c}^{{{{{{{{\rm{inh}}}}}}}}} 	\to {A}_{c}^{{{{{{{{\rm{inh}}}}}}}}}+\eta ({x}_{{{{{{{{\rm{post}}}}}}}}}-\alpha )\,{{{{{{{\rm{with}}}}}}}}\,{{{{{{{\rm{each}}}}}}}}\,{{{{{{{\rm{pre}}}}}}}}{{{{{{{\rm{synaptic}}}}}}}}\,{{{{{{{\rm{spikes}}}}}}}}\\ {A}_{c}^{{{{{{{{\rm{inh}}}}}}}}} 	\to {A}_{c}^{{{{{{{{\rm{inh}}}}}}}}}+\eta {x}_{{{{{{{{\rm{pre}}}}}}}}}\,{{{{{{{\rm{with}}}}}}}}\,{{{{{{{\rm{each}}}}}}}}\,{{{{{{{\rm{post}}}}}}}}{{{{{{{\rm{synaptic}}}}}}}}\,{{{{{{{\rm{spikes}}}}}}}}$$

where *η* is the learning rate, *α* = 2 ⋅ *r*_target_ ⋅ *τ*_STDP_ is the depression factor, where *τ*_STDP_ = 20 ms is the STDP time constant, and *r*_target_ = 5Hz is a constant parameter that defines the target postsynaptic firing rate. In simulations, the initial values of *w*_ISP_ is set to zero. *A*_inh_ is initialized to 0.35 nS.

#### ISP with STP

In our simulations, ISP is combined with STP in some cases at the inhibitory synapses. In these cases, the total synaptic weight *w*_inh_ is computed as the product of the STP and ISP weight components at the time of the postsynaptic spike ${w}_{{{{{{{{\rm{inh}}}}}}}}}={w}_{{{{{{{{\rm{STP}}}}}}}}}^{{{{{{{{\rm{inh}}}}}}}}}\cdot {w}_{{{{{{{{\rm{ISP}}}}}}}}}$ while the excitatory weight was given by ${w}_{{{{{{{{\rm{exc}}}}}}}}}={w}_{{{{{{{{\rm{STP}}}}}}}}}^{{{{{{{{\rm{exc}}}}}}}}}$.

### Simulations

#### Input signals and connectivity

To model the neural responses we used 8 independently generated traces of low-pass filtered, half-wave rectified white noise signals. Each of the 8 independent channels represents a signal pathway, and consists of 100 excitatory neurons and 25 inhibitory neurons, giving a total of 1000 presynaptic neurons^22^. All presynaptic neurons synapse onto a single postsynaptic neuron with a total of 1000 synapses, 800 excitatory and 200 inhibitory.

As in previous studies^22^ for each of the 8 channels, we generated its time-varying rates iteratively as ${\hat{s}}_{k}(t+dt)=\xi -(\xi -{\hat{s}}_{k}(t))\cdot {e}^{-\frac{dt}{{\tau }_{s}}}$ where ${\hat{s}}_{k}$ is the *k*-th signal, *ξ* ∈ [−0.5, 0.5] is drawn from a uniform distribution, *dt* = 0.1 ms is the simulation time step, and the filtering time constant is *τ*_*s*_ = 50 ms. We normalized all rates to a preferred firing rate of 100 Hz, and negative values were remove and replaced with a background activity level of 5Hz.

These traces represent the firing rates across time of each of the 8 input signal channels (see examples in Fig. S2b). We used these rates as seeds to generate Poisson spike trains for each of the eight channels. These inputs were used in the simulations shown in Figs. 1, 2 and S6.

#### Developmental and fixed STP

When simulating dev-STP, we first found the STP parameters whose paired-pulse ratio (PPR, i.e. EPSP_2_/EPSP_1_) best matched experimental data^8^. To this end, we started with STP parameters which give strong depression and strong facilitation^79^. Next, we conducted a parameter sweep of the STP parameters from strong depression to strong facilitation using a dense linear space between these two conditions. We then simulated 50 Poisson input spike trains at 35Hz^8^, calculated the average PPRs of each train for all STP parameters. We then used the STP parameter values that best matched those observed experimentally^8^ for our simulations. These parameter values are summarized in Table 2.

#### Calibrating the parameters for dev-STP

Using the STD and STF parameters given in Table 2, we then calculated a set of 3600 parameter values spaced logarithmically between the STD and the STF parameter values. Below we use *d* to denote the exact developmental stage, i.e. *d* ∈ {1, 2, ⋯, 3600}. Log interpolation was used instead of linear interpolation because a marginal change towards facilitation generates a higher marginal change in PPR when closer to facilitation than to depression. For each of the 3600 STP parameter values, each time we changed STP parameters, we normalized the STP magnitude parameter *A* to equal

5 $${A}_{c,d}^{{{{{{{{\rm{exc}}}}}}}}} 	=\frac{{A}_{c,d = 1}^{{{{{{{{\rm{exc}}}}}}}}}}{{u}_{d}(t=0)\cdot {R}_{d}(t=0)}\\ 	=\frac{{A}_{c,d = 1}^{{{{{{{{\rm{exc}}}}}}}}}}{{U}_{d}}$$

where ${A}_{c,d = 1}^{{{{{{{{\rm{exc}}}}}}}}}$ is the baseline excitatory weight as defined by the each input channel (see "Excitatory and Inhibitory tuning curves” below). The subscript *c* represents the input channel number, and the subscript *d* ∈ {1, 2, ⋯, 3600} are the parameter values during specific developmental stages. This normalization fixed the amplitude of the first PSP to the same value, regardless of the STP parameters, thus keeping the baseline weight of excitatory synapses the same throughout development during the simulation (see below for alternative normalizations). Note that the initial value of *u* is set to *U*, the initial value of *R* is set to 1, and the total excitatory weight for a first presynaptic spike is given by

6 $${w}_{{{{{{{{\rm{exc}}}}}}}}}(t=0) 	={w}_{{{{{{{{\rm{STP}}}}}}}}}^{{{{{{{{\rm{exc}}}}}}}}}(t=0)\\ 	={A}_{c,d = 1}^{{{{{{{{\rm{exc}}}}}}}}}{R}_{d}(t=0){u}_{d}(t=0)\\ 	=\frac{{A}_{c,d = 1}^{{{{{{{{\rm{exc}}}}}}}}}}{{U}_{d}}\cdot 1\cdot {U}_{d}\\ 	={A}_{c,d = 1}^{{{{{{{{\rm{exc}}}}}}}}}$$

regardless of the STP parameters, thus the baseline excitatory weight is invariant across development in our simulations.

To start the dev-STP simulation, we used the baseline STD parameters given in Table 2 at the beginning of the simulation, and slowly changed the parameters from depressing to facilitating at excitatory synapses. Toward this end, we averaged the postsynaptic neuron’s firing rate over a 500 ms window and monitored how often it exceeded the ISP target rate of 5Hz by way of a variable *x*_exceed_ that was updated as follows

7 $${x}_{{{{{{{{\rm{exceed}}}}}}}}}=\left\{\begin{array}{ll}{x}_{{{{{{{{\rm{exceed}}}}}}}}}+\lceil \frac{{r}_{{{{{{{{\rm{post}}}}}}}}}}{{r}_{{{{{{{{\rm{target}}}}}}}}}}\rceil \quad &{{{{{{{\rm{if}}}}}}}}{r}_{{{{{{{{\rm{post}}}}}}}}}\ge {r}_{{{{{{{{\rm{target}}}}}}}}}\\ {x}_{{{{{{{{\rm{exceed}}}}}}}}}-1\hfill &{{{{{{{\rm{if}}}}}}}}{r}_{{{{{{{{\rm{post}}}}}}}}} < {r}_{{{{{{{{\rm{target}}}}}}}}}\end{array}\right.$$

where *r*_post_ is the postsynaptic firing rate and *r*_target_ is the ISP target rate (see above). We increment STP to the next set of more facilitating STP parameters when *x*_exceed_ ≤ 0. In other words, the STP parameters are incremented only when the postsynaptic firing rate is equal to or below the ISP target rate for a sufficient period of time, i.e., a time that is proportional to the degree to which the postsynaptic firing rate has exceeded the target rate in the recent past. Changing the excitatory STP to a more facilitating state raises the postsynaptic firing rate, which increases *x*_exceed_, thus preventing further facilitating changes in STP until inhibitory synaptic weights strengthen and subsequently decrease the postsynaptic firing rate to the target rate, and the cycle starts over. Eventually, the STP parameter values reach the final (experimentally observed^8^) STF parameter values (given in Table 2).

For both the fixed-STF and fixed-STD simulations, STP parameters at all excitatory synapses were set to depression and facilitation (Table 2), respectively, for the duration of the simulation.

Further, we quantified the level of “pathological activity” in all three models as the cumulative difference between the observed firing rate and the target firing rate for all input channels (Fig. 2g.i). We also considered the variability of firing rates, i.e. the coefficient of variation (standard deviation divided by the mean) of the firing rates averaged across 10 s bins using a sliding window (Fig. 2g.ii).

To ease the comparison of our results across figures we provide a supp. figure highlighting the key results of dev-STP Fig. S1.

#### Variants of developmental STP model

We conducted additional simulations to better demonstrate the behavior of the model under different conditions (see summary of model variants in Table 3). Note that models without the proposed combination of STP or ISP would result in high firing rates and potentially unstable dynamics (Fig. 1e) while lacking some of the functional properties of dev-STP (Figs. S18 and S19).

**Table 3:** Summary table of the various variants of dev-STP models and how these impact the different parameters.

Model name	*A*	*U*	*A* ⋅ *U* (first PSP)	Inh. r.f. dev.
0. Standard dev-STP	Increasing	Decreasing	Constant	Yes
1. Fixed *A* to that of STD	Decreasing	Decreasing	Decreasing	Yes
2. Facilitation-Depression STP	Increasing	Decreasing	Constant	Yes
3. Norm. *A* to steady-state 5Hz	Decreasing	Decreasing	Decreasing	Yes
4. Norm. *A* to steady-state 10Hz	Decreasing	Decreasing	Decreasing	No
5. Predefined STP changes	Decreasing	Decreasing	Constant	Yes
6. Stochastic dev-STP	Increasing	Decreasing	Constant	Yes

We provide a summary of how the different short-term dynamics look like in Fig. S3 and how the different model parameters change across simulated development in Fig. S4.

1. Fixed *A* to that of STD: First, we considered a simpler model in which the synaptic scaling factor, *A* is fixed throughout development and set to the initial value (i.e., that of STD) (Fig. S7). The results are qualitatively the same as for the dev-STP, showing slightly faster STP development due to overall weaker scaling factor (compare Fig. S7e with Fig. 2e).

2. Facilitation-depression STP: Next, we considered another variant of the dev-STP model with adult STF that captures the degree of facilitation-depression observed experimentally in adult animals in primary sensory cortices^8^ (see Fig. S3; see Table 4 for the parameters used). In this model (Fig. S8) we get qualitatively the same results as the original dev-STP model which uses stronger STF in adult. To compensate for the weaker facilitation we observe a slightly faster rate of change in the STP parameters over development (compare Fig. S8e with Fig. 2e).

**Table 4:** STP parameter values used for the facilitation-depression STP model.

Synaptic dynamics	Connection	*D* (s)	*F* (s)	*U*	*f*	PPR
Depression	exc. (young); inh.	0.3134	0.0798	0.3917	0.062	0.70
Facilitation–depression^79^	exc. (adult)	0.2	0.2	0.25	0.3	1.42

Paired-pulse ratio (PPR) is given by dividing the second postsynaptic response by the first.

3. Norm. *A* to steady-state 5Hz: Next, we tested a model variant in which we normalized the steady-state PSP amplitudes when using a 5 Hz presynaptic Poisson input (Fig. S9) instead of normalizing to the first PSP. STP parameters in this dev-STP model were modified over development as described above. In this variant, the fixed-STF model displayed a lower initial firing rate than that of the standard model (Fig. S9b), failing to reach the ISP target rate and experimentally observed firing rates in young animals^28–31^. Receptive field development in this variant is otherwise qualitatively similar to our dev-STP model, if somewhat more slowly (Fig. S9g).

4. Norm. *A* to steady-state 10Hz: In addition, we also normalized the steady-state PSP of both STD and STF to be equal when using a 10Hz (instead of 5Hz as in the standard model) presynaptic Poisson input (Fig. S10). In this case, STF was weakened enough that fixed-STF in young animals exhibited firing rates near the ISP target rate as observed experimentally^28–31^. However, because of weakened STF, which caused the firing rate to stay below the target firing rate for ISP, which leads to a lack of fine-tuned tuning curves over development (Fig. S10f–h). Note that this is purely due to how the ISP learning rule is defined as commonly done in the field, not our developmental model.

5. Predefined STP changes: We also tested a variant of our model in which the developmental shift from STD in young neurons to STF in adult neurons was not activity-dependent. Instead, we altered the dev-STP model to a model in which STP changes occurred at fixed intervals of 3seconds (Fig. S11e). If these changes occur too frequently, unstable dynamics unfolded so some fine tuning of how often STP changes is required. This model variant also produced qualitatively similar results to our standard dev-STP model (compare Fig. S11 and Fig. 2).

6. Dev-STP with stochastic release: Finally, we tested a variant of the dev-STP model with stochastic excitatory and inhibitory synapses. Stochastic release using an uniform distribution and the release probability *u* to sample release events. This model variant produces results qualitative similar to the main dev-STP model and also shows reduced jitter over development (Fig. S17).

#### Excitatory and inhibitory tuning curves

To calculate the excitatory and inhibitory tuning curves, we monitored the excitatory and inhibitory conductances for each of the 8 input channels separately, and calculated the respective currents using

8 $${I}_{k}^{{{{{{{{\rm{exc}}}}}}}}}(t) 	={g}_{k}^{{{{{{{{\rm{exc}}}}}}}}}(t)({E}_{{{{{{{{\rm{exc}}}}}}}}}-V(t))\\ {I}_{k}^{{{{{{{{\rm{inh}}}}}}}}}(t) 	={g}_{k}^{{{{{{{{\rm{inh}}}}}}}}}(t)({E}_{{{{{{{{\rm{inh}}}}}}}}}-V(t))+{g}_{{{{{{{{\rm{leak}}}}}}}}}({V}_{{{{{{{{\rm{rest}}}}}}}}}-V(t))/K$$

where ${I}_{k}^{{{{{{{{\rm{exc}}}}}}}}}(t)$ and ${I}_{k}^{{{{{{{{\rm{inh}}}}}}}}}(t)$ are the excitatory and inhibitory currents and ${g}_{k}^{{{{{{{{\rm{exc}}}}}}}}}(t)$ and ${g}_{k}^{{{{{{{{\rm{inh}}}}}}}}}(t)$ are the excitatory and inhibitory conductances of the *k*-th channel at time *t*, respectively^22^. *E*_exc_ and *E*_inh_ are the excitatory and inhibitory reversal potentials, respectively. *V*(*t*) is the postsynaptic membrane potential at time *t*, *g*_leak_ is the leaky conductance, and *V*_rest_ is the resting membrane potential. After calculating the excitatory and inhibitory currents for each channel at all time points, we averaged the excitatory and inhibitory currents across 10seconds to generate the tuning curves shown in the figures.

#### Output response dynamics across development

To measure how the neuron output response changed over the course of simulated development, we stopped the dev-STP simulation (Fig. 2) at 10 s, 500 s, 1000 s, 2,000 s, 10,000 s, and 30,000 s simulated time and examined the response dynamics of the model neuron. For each snapshot, we ran 50 step current trials with frozen parameters and compared the average firing rates of the dev-STP scenario with those of the fixed-STD and fixed-STF scenarios (Fig. 4b).

To investigate how input tuning changed over development, we calculated the cross correlations between the input and output rates for each of the 8 channels^22^. We obtained the correlation range by subtracting the minimum from the maximum correlation and normalized the range by dividing by the mean correlation of all channels with the output (Fig. 4d).

#### Signal transmission across development

To investigate signal transmission across development, we presented a 250 ms long 150 Hz input stimulus to the preferred input channel every 100seconds of the dev-STP simulation (Fig. 2). We analyzed the output firing rates during the first 50 ms after stimulus onset (phasic period) and the remaining 200 ms afterwards (tonic period); Fig. 5b, c). We also tested a double step-input stimulus, two 250 ms 150 Hz input stimuli separated by 250 ms of spontaneous activity (Fig. 5d, e).

#### Temporal precision simulations

We compared the temporal precision of postsynaptic spikes in our model with experimental observations^26^. To this end, we stimulated the preferred channel (5) of the output neuron with a 200 ms step current, imitating a pure tone in the preferred frequency in the auditory cortex^26^. To quantify the temporal precision of the response, we calculated the standard deviation of the delay between the stimulus onset and the first postsynaptic spike, denoted as the jitter^26^. To allow comparison across different firing rates, we also calculated a normalized jitter, i.e., the jitter’s coefficient of variation. The normalized jitter was compared for different preferred channel stimulus strengths as well as for varying spontaneous activity levels (Fig. 6c–e).

#### Short-term memory traces

To test for short-term memory properties, we used two simulation protocols. In the "memory preloaded” trials, we stimulated the neuron with a 300 ms long 150 Hz steady-state stimulus (a memory) in the preferred channel. All remaining channels received spontaneous rates at 5Hz. After the memory preloading period, the preferred channel input received spontaneous firing rate inputs for a 300 ms delay period, followed by a weaker, 100 ms long 50 Hz "recall cue” stimulus. For "control” trials, the input channels of the neuron only received the 100ms recall cue, to the preferred channel, without preloading.

We then compared the firing rates during recall between the memory preloaded and control trials, to study the “silent” short-lasting memory effects in our model. We tested this throughout simulated development, by freezing the dev-STP simulation every 50s and simulating 500 trials of the memory preloaded simulations and 500 trials of the control simulations.

#### Simulator

Simulations were conducted in Python using Brian Simulator 2.

### Reporting summary

Further information on research design is available in the Nature Research Reporting Summary linked to this article.

## Supplementary information

Supplementary Information

Reporting Summary

## Data Availability

The data analyzed in this study are included in a previous study^26^ (and its supplementary information files).

## Code availability

Code to reproduce our key findings is available at github.com/djia/dev-stp.
