# Supplementary material for: Developmental depression-to-facilitation shift controls excitation-inhibition balance
Source: Commun Biol. 2022 Aug 25;5:873. doi: 10.1038/s42003-022-03801-2 (PMC9411206; doi:10.1038/s42003-022-03801-2)
Supplement: Supplementary file 1 — Supplementary Information [file 42003_2022_3801_MOESM1_ESM.pdf]

Supplementary figures

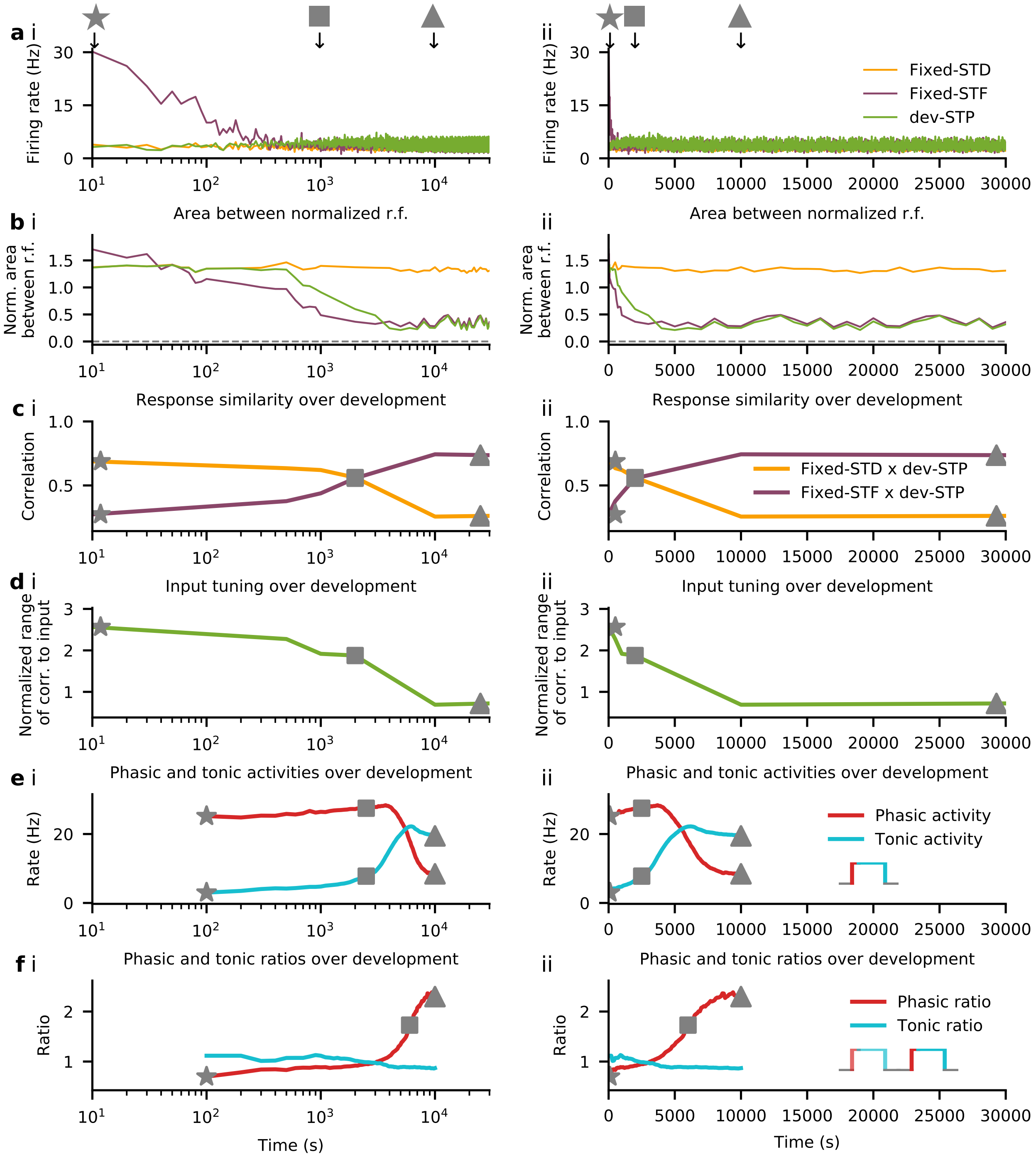

Figure S1. Comparison of key devSTP results. Results are shown in both log (left) and normal (right) time axis. (**a**) Model firing rates (cf. Fig. 2). (**b**) Area between exc-inh receptive fields (cf. Fig. 2). (**c**) Activity correlation analysis between fixed STP models and dev-STP (cf. Fig. 4). (**d**) Input tuning over development (cf. Fig. 4). (**e**) Phasic and tonic activities over development (cf. Fig. 5). (**f**) Phasic and tonic ratios over development (cf. Fig. 5). The time points that are highlighted in the respective (main) figures were chosen to demonstrate points of interest in the model behaviour in early (star), mid (square) and late (triangle) periods during simulated development.

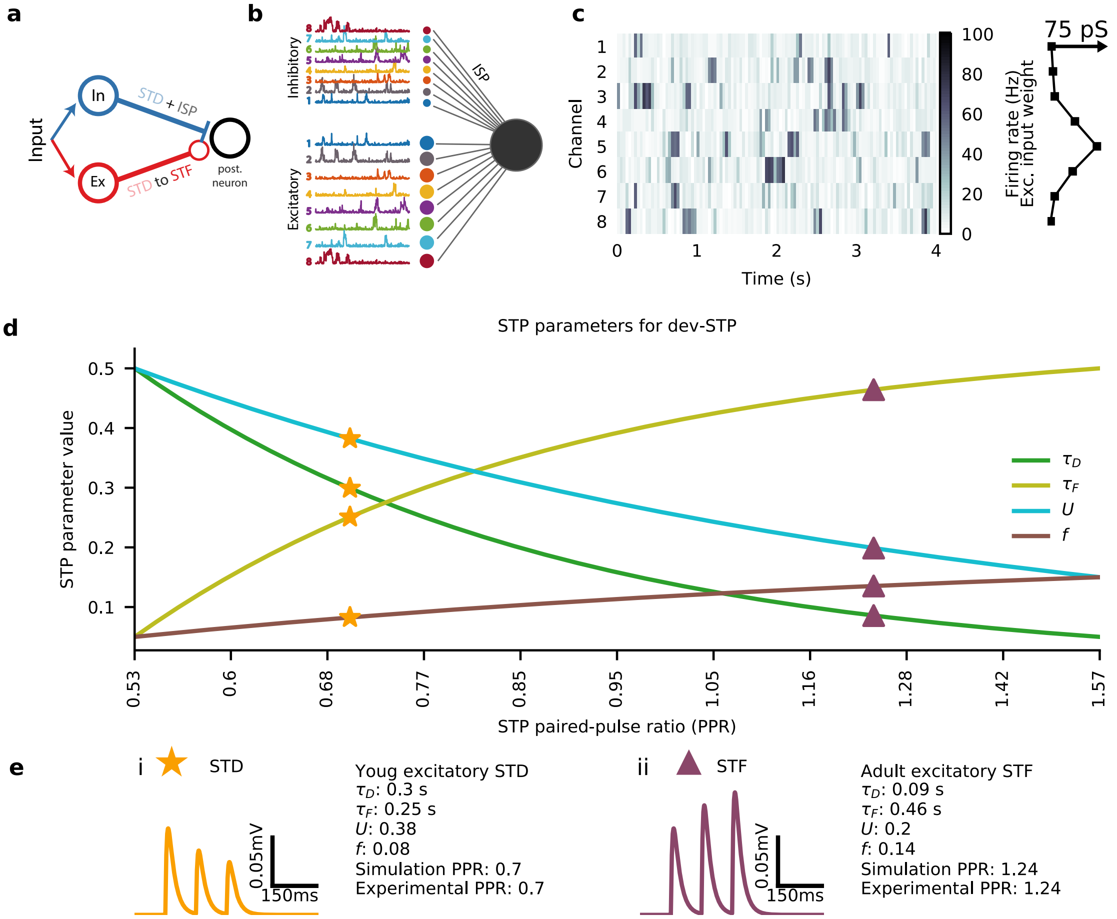

Figure S2. Details of the cortical circuit and plasticity models. **(a)** Schematic of a single channel feedforward circuit with correlated excitatory and inhibitory input, and the respective forms of plasticity. **(b)** Feedforward neural circuit with 8 channels and correlated excitatory and inhibitory inputs. **(c)** Left: example of input given to the 8 channel feedforward neural circuit; right: excitatory tuning curve strength for each of the 8 channels. **(d)** Each of the four STP parameters, τ_D, τ_F, U, and f resulting in different paired-pulse ratios (PPRs) (Table 2). Parameters matching the young (orange star) and adult (purple triangle) STP PPRs as used in the dev-STP model are highlighted. **(e)** Example postsynaptic potential traces for the STP parameter values of both young (i) and adult animals (ii; cf. **d**).

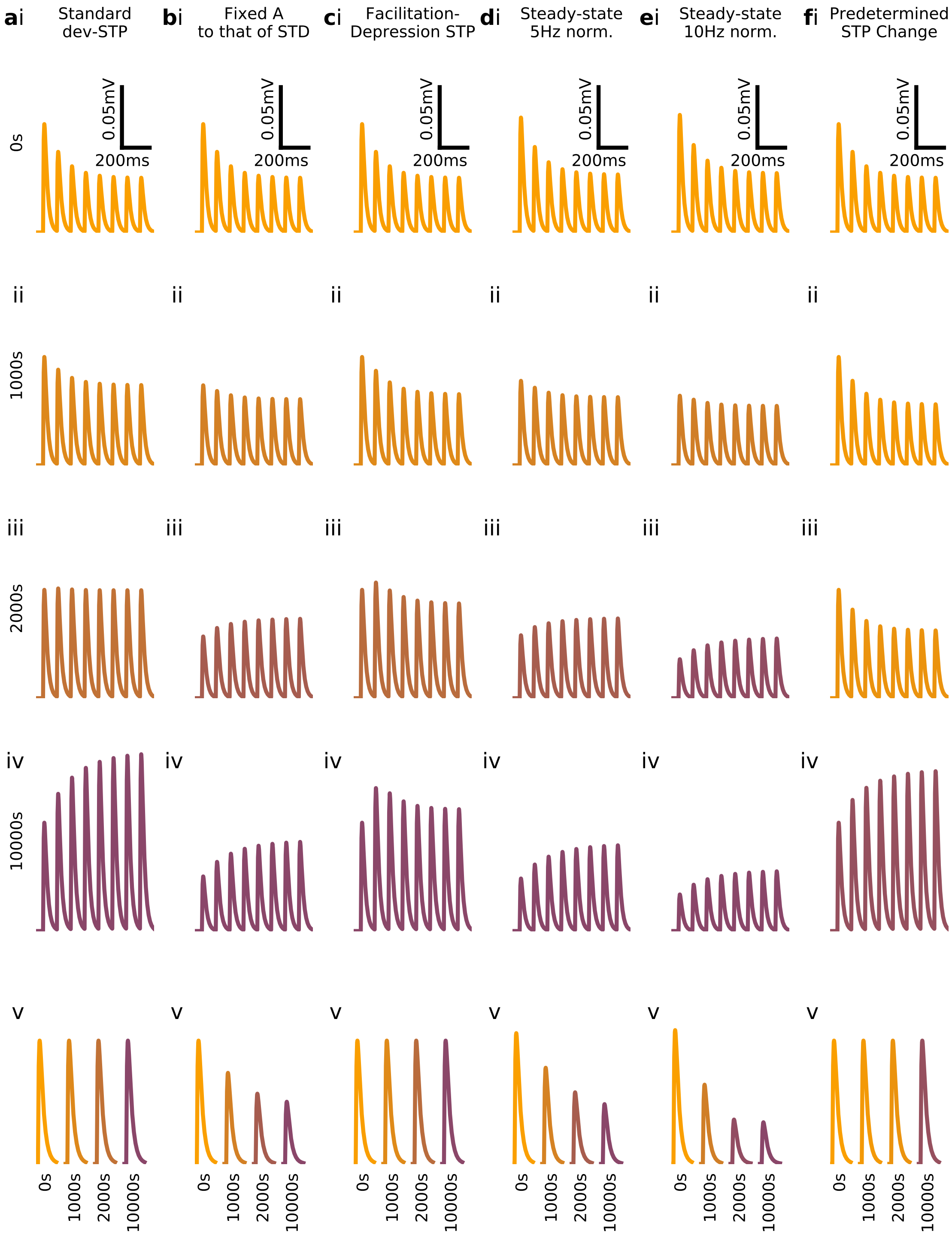

Figure S3. Example EPSPs across development for all models. (**a**, **b**, **c**, **d**, **e**) example EPSPs for different models tested at various times during the simulation: at 0s (i), 1000s (ii), 2000s (iii), and 10000s (iv); (v) compares the baseline (first) EPSPs for a given model across various times of the simulation. Colors represent STP type: more orange is closer to STD, more purple is closer to STF.

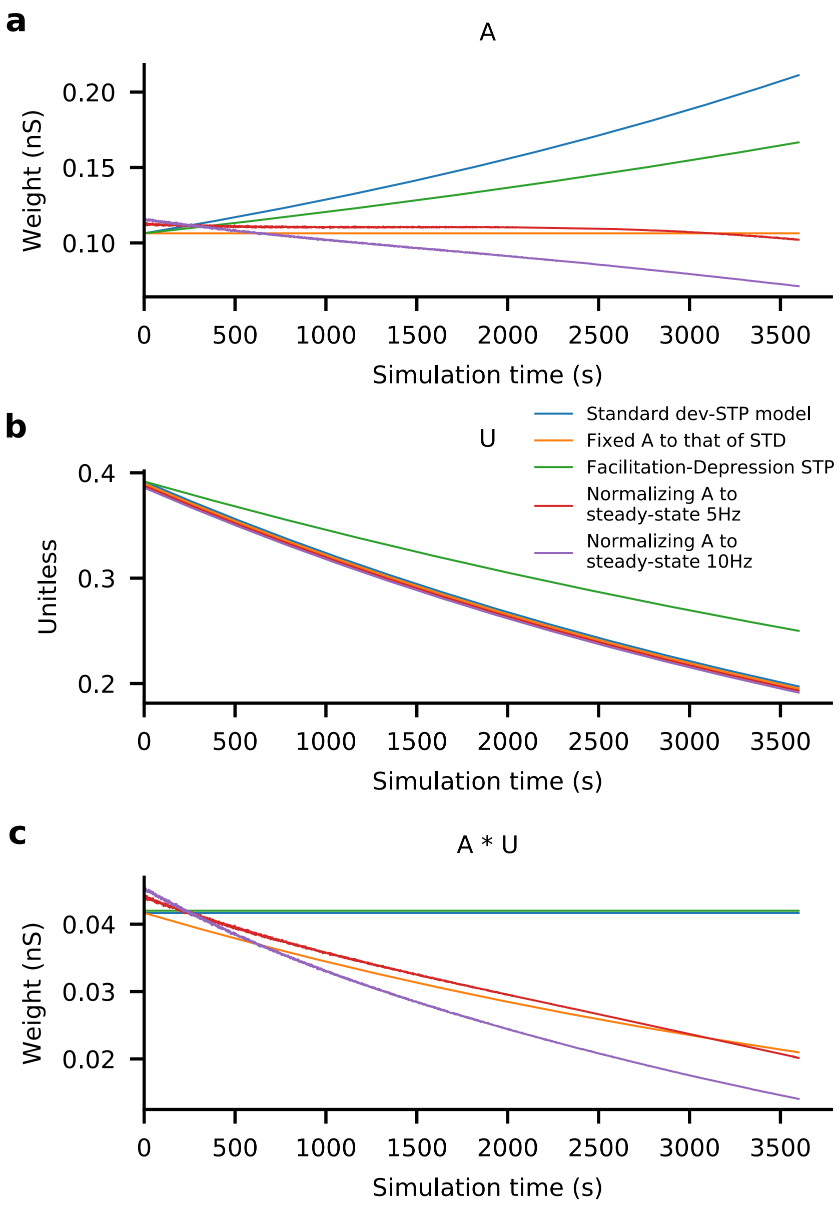

Figure S4. STP parameter evolution across development for model variants. (**a**) Evolution of the A (postsynaptic scaling constant) parameter across development. (**b**) Evolution of U (baseline release probability) parameter across development. (**c**) Evolution of the A * U across development, which represents baseline weight.

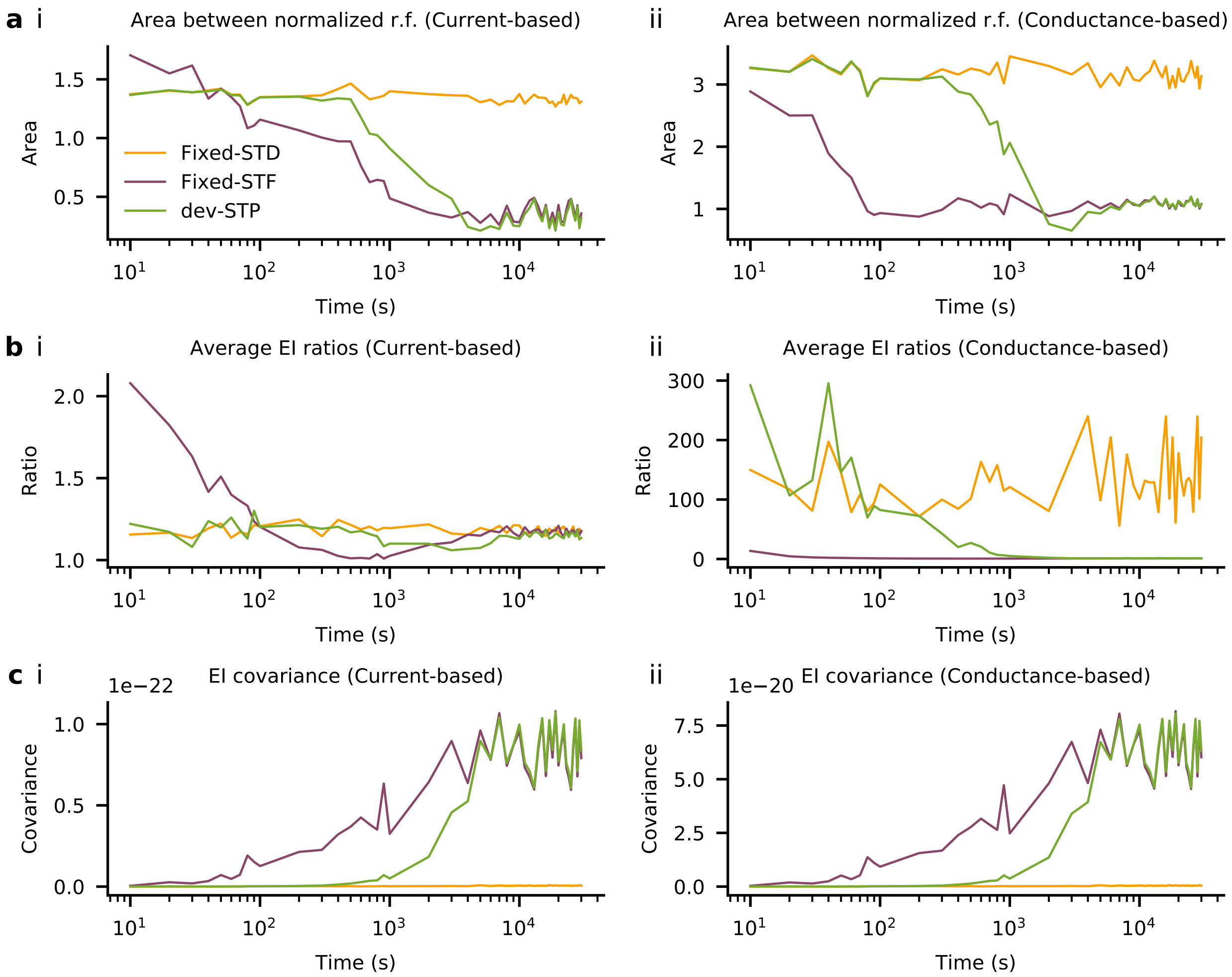

Figure S5. Comparison of multiple excitation-inhibition balance measures. (**a**) Area between exc. and inh. receptive fields for both current-based (i; as in Fig. 2f) and conductance-based inputs (ii). A area of zero represents perfect exc-inh balance. (**b**) Simple ratio between exc. and inh. current-based (i) and conductance-based inputs (ii). A ratio close to 1 represents a good exc-inh balance. (**c**) Covariance between exc. and inh. current-based (i) and conductance-based inputs (ii). Covariance should increase as inh. starts matching the excitatory receptive fields. Note that the area measure is better at capturing the match between exc. and inh. receptive fields (c.f. Fig. 2h-j).

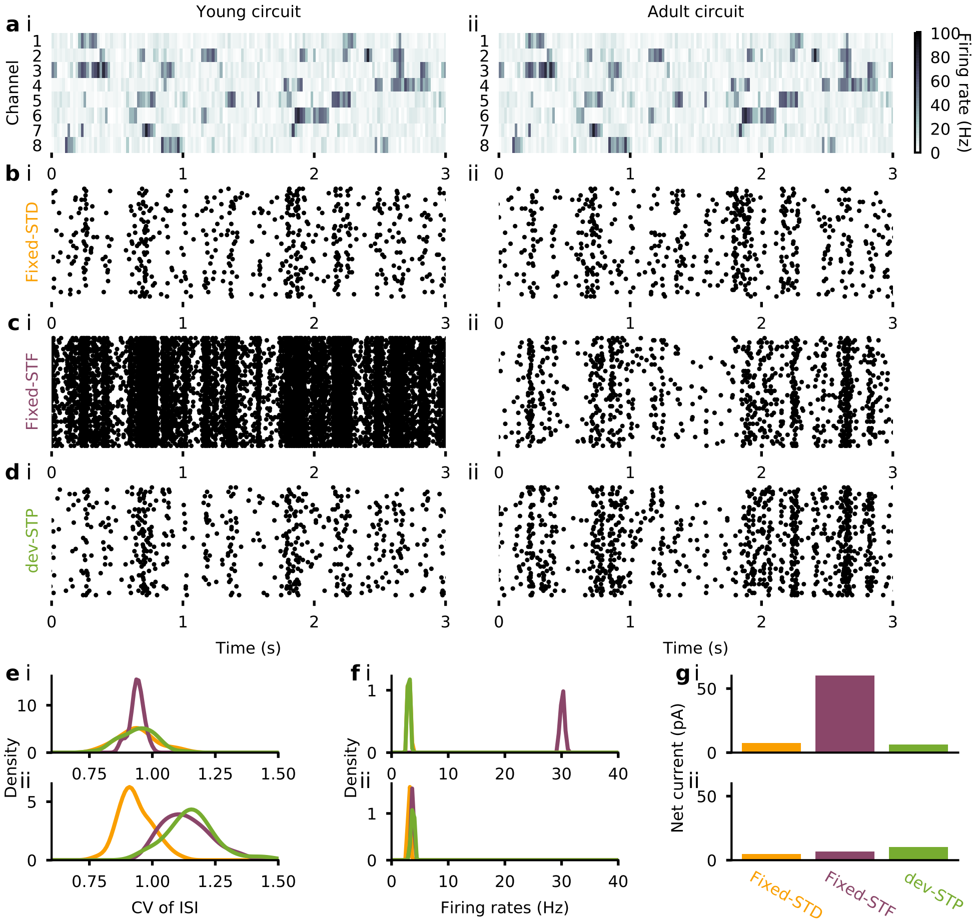

Figure S6. Developmental STP shapes firing statistics. (**a**) Input activity for each of the 8 channels over 3 seconds. Activity at the start of simulated development (i, young condition) and after 8 hours of simulation (ii, adult condition) as in Fig. 2; color code represents firing rate of input. (**b-d**) Raster plot of receiver neuron for fixed-STD model (b), fixed-STF (c) and developmental STP model (d). (**e-g**) Summary statistics of the three models (as in b-d) for both young (i) and adult conditions (ii). (**e**) Coefficient of variation of the inter-spike intervals. (**f**) Average firing rates of the receiver neuron over 50 trials. (**g**) Average net current of the receiver neuron.

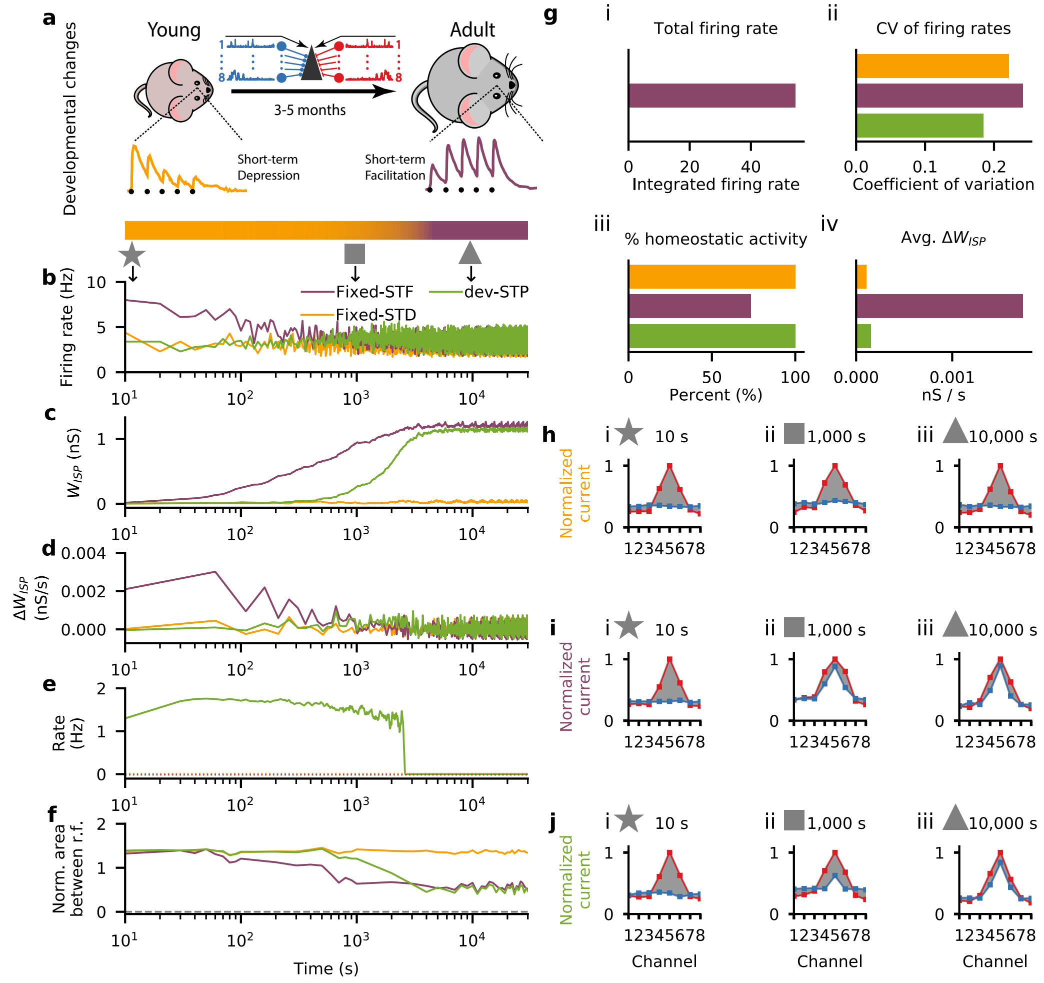

Figure S7. Developmental STP model with constant A fixed to that of STD. (**a**) Schematic of our developmental short-term plasticity (STP) model (cf. Fig. S2); top: young and adult STP (as in Fig. 1); bottom: gradual changes in STP from depressing to facilitating dynamics (orange and purple respectively, in log-scale as in b-f). (**b**-**f**) Different variables of the model across simulated development for three different models: fixed short-term depression (fixed-STD, orange), fixed short-term facilitation (fixed-STF, purple) and developmental model with gradual changes in STP (dev-STP, green line). Note x-axis on log-scale. (**b**) Receiver neuron firing rate. (**c**) Mean inhibitory weight. (**d**) Mean changes in the weight of the inhibitory synaptic afferents. (**e**) Rate of STP change (note that both fixed-STF and STD remain fixed, shown as dashed lines). (**f**) Area between normalised excitatory and inhibitory tuning curves (cf. h-j) during the course of simulated development. A normalised area close to 0 represents a perfectly balanced neuron (cf. S5 with other EI balance metrics). (**g**) Additional statistics for the three models. (i) Total neuronal activity calculated using the area between the firing rate in (b) and the desired target rate of 5 Hz. (ii) Average coefficient of variation of the firing rates across simulated development (cf. (b)). (iii) Percent of time spent under homeostasis (i.e. at the desired firing rate; cf. (b)). (iv) Average change in inhibitory weights (cf. (d)). (**h**-**j**) Snapshots of excitatory and inhibitory tuning curves across three points in simulated development: 10s (star), 1000s (square) and 10 000s (triangle). Shaded gray area represents difference between excitatory and inhibitory tuning curves (cf. (f)). (**h**-**j**) Excitatory (red) and inhibitory (blue) postsynaptic tuning curve for the fixed-STD (h), fixed-STF (i) and dev-STP models (j).

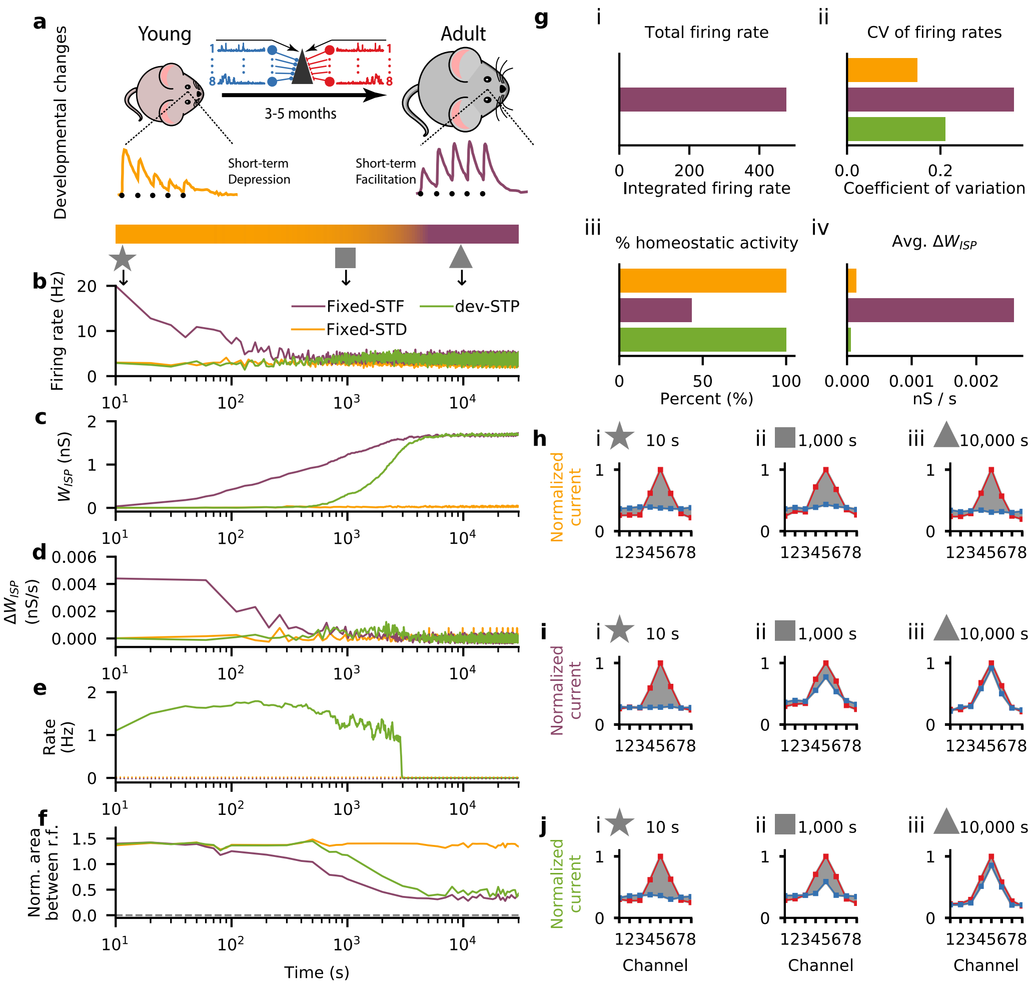

Figure S8. Developmental STP model with combined depression-facilitation in the adult STP condition. (**a**) Schematic of our developmental short-term plasticity (STP) model (cf. Fig. S2); top: young and adult STP (as in Fig. 1); bottom: gradual changes in STP from depressing to facilitating dynamics (orange and purple respectively, in log-scale as in b-f). (**b**-**f**) Different variables of the model across simulated development for three different models: fixed short-term depression (fixed-STD, orange), fixed short-term facilitation (fixed-STF, purple) and developmental model with gradual changes in STP (dev-STP, green line). Note x-axis on log-scale. (**b**) Receiver neuron firing rate. (**c**) Mean inhibitory weight. (**d**) Mean changes in the weight of the inhibitory synaptic afferents. (**e**) Rate of STP change (note that both fixed-STF and STD remain fixed, shown as dashed lines). (**f**) Area between normalised excitatory and inhibitory tuning curves (cf. h-j) during the course of simulated development. A normalised area close to 0 represents a perfectly balanced neuron. (**g**) Additional statistics for the three models. (i) Total neuronal activity calculated using the area between the firing rate in (b) and the desired target rate of 5 Hz. (ii) Average coefficient of variation of the firing rates across simulated development (cf. (b)). (iii) Percent of time spent under homeostasis (i.e. at the desired firing rate; cf. (b)). (iv) Average change in inhibitory weights (cf. (d)). (**h**-**j**) Snapshots of excitatory and inhibitory tuning curves across three points in simulated development: 10s (star), 1000s (square) and 10 000s (triangle). Shaded gray area represents difference between excitatory and inhibitory tuning curves (cf. (f)). (**h**-**j**) Excitatory (red) and inhibitory (blue) postsynaptic tuning curve for the fixed-STD (h), fixed-STF (i) and dev-STP models (j).

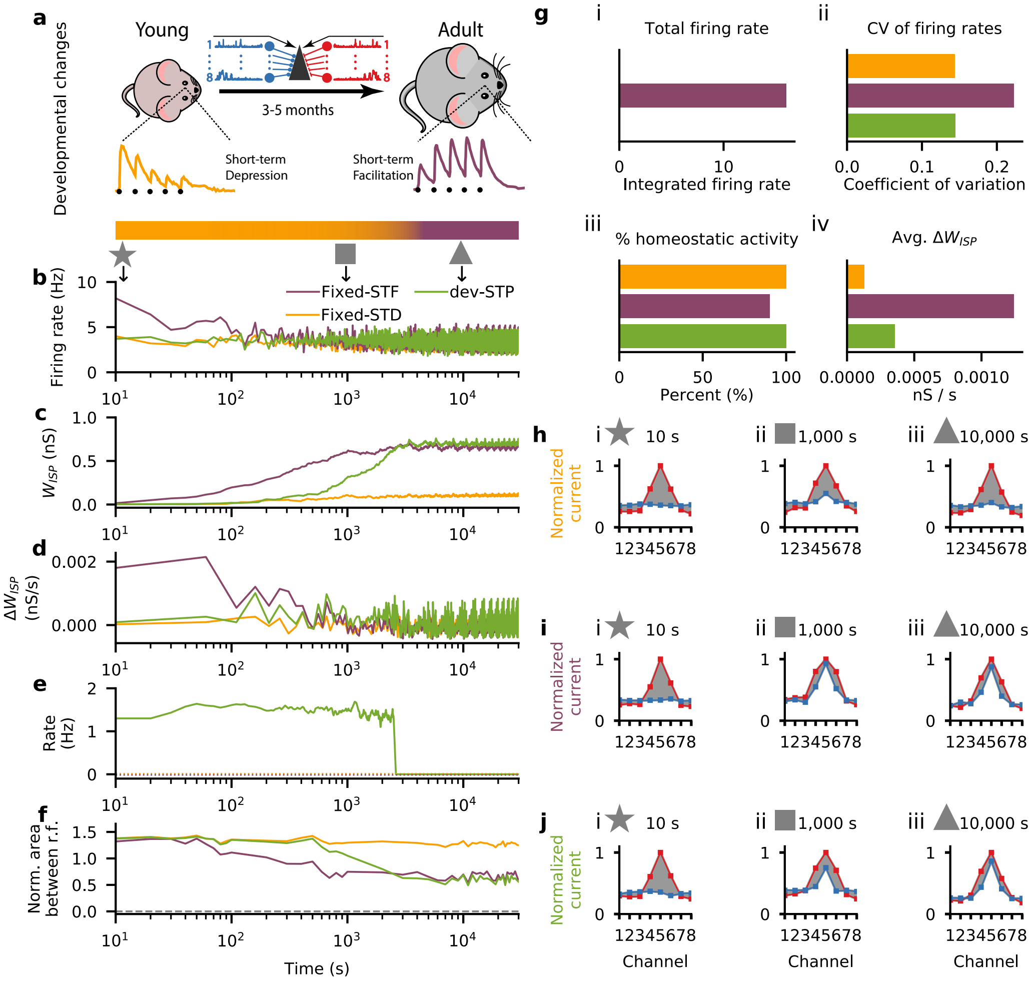

Figure S9. Developmental STP model with depression and facilitation normalized to the steady-state firing rate at 5Hz input. (**a**) Schematic of our developmental short-term plasticity (STP) model (cf. Fig. S2); top: young and adult STP (as in Fig. 1); bottom: gradual changes in STP from depressing to facilitating dynamics (orange and purple respectively, in log-scale as in b-f). (**b**-**f**) Different variables of the model across simulated development for three different models: fixed short-term depression (fixed-STD, orange), fixed short-term facilitation (fixed-STF, purple) and developmental model with gradual changes in STP (dev-STP, green line). Note x-axis on log-scale. (**b**) Receiver neuron firing rate. (**c**) Mean inhibitory weight. (**d**) Mean changes in the weight of the inhibitory synaptic afferents. (**e**) Rate of STP change (note that both fixed-STF and STD remain fixed, shown as dashed lines). (**f**) Area between normalised excitatory and inhibitory tuning curves (cf. h-j) during the course of simulated development. A normalised area close to 0 represents a perfectly balanced neuron. (**g**) Additional statistics for the three models. (i) Total neuronal activity calculated using the area between the firing rate in (b) and the desired target rate of 5 Hz. (ii) Average coefficient of variation of the firing rates across simulated development (cf. (b)). (iii) Percent of time spent under homeostasis (i.e. at the desired firing rate; cf. (b)). (iv) Average change in inhibitory weights (cf. (d)). (**h**-**j**) Snapshots of excitatory and inhibitory tuning curves across three points in simulated development: 10s (star), 1000s (square) and 10 000s (triangle). Shaded gray area represents difference between excitatory and inhibitory tuning curves (cf. (f)). (**h**-**j**) Excitatory (red) and inhibitory (blue) postsynaptic tuning curve for the fixed-STD (h), fixed-STF (i) and dev-STP models (j).

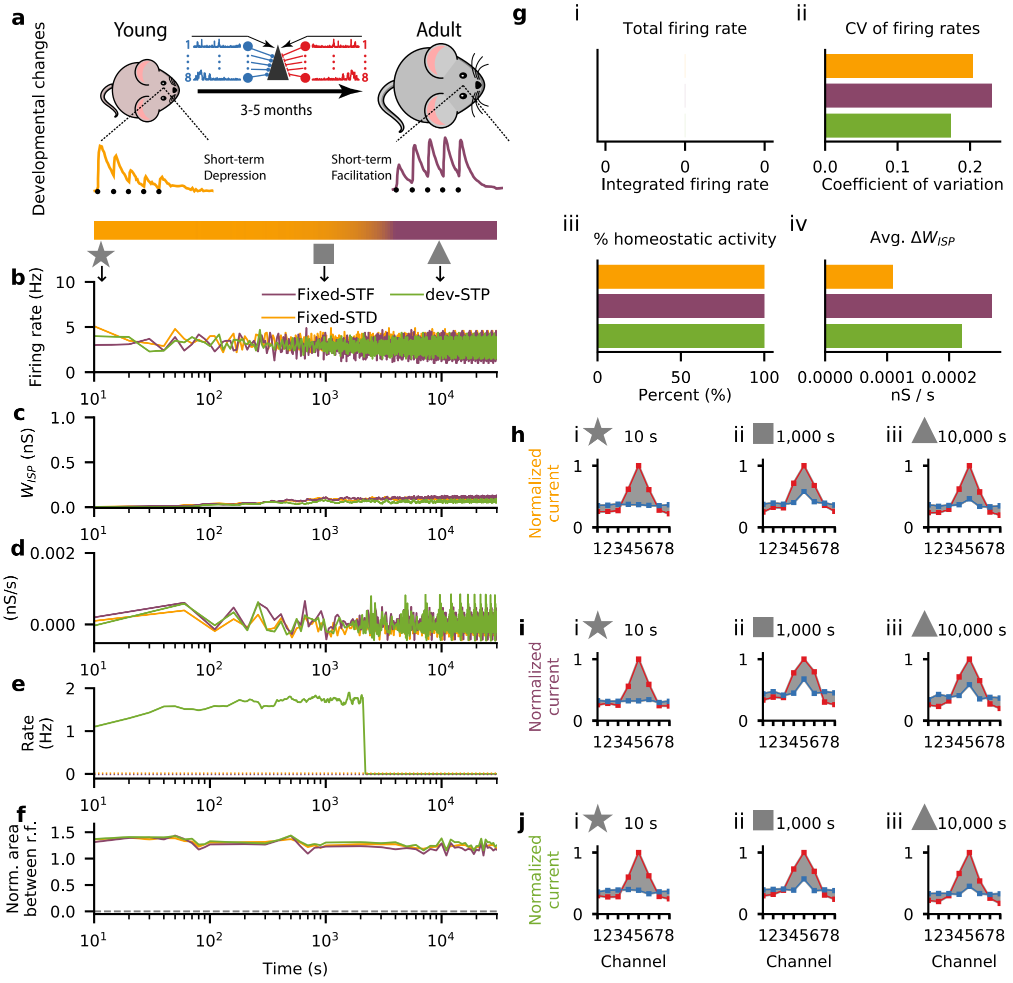

Figure S10. Developmental STP model with depression and facilitation normalized to the steady-state firing rate at 10Hz input. (**a**) Schematic of our developmental short-term plasticity (STP) model (cf. Fig. S2); top: young and adult STP (as in Fig. 1); bottom: gradual changes in STP from depressing to facilitating dynamics (orange and purple respectively, in log-scale as in b-f). (**b-f**) Different variables of the model across simulated development for three different models: fixed short-term depression (fixed-STD, orange), fixed short-term facilitation (fixed-STF, purple) and developmental model with gradual changes in STP (dev-STP, green line). Note x-axis on log-scale. (**b**) Receiver neuron firing rate. (**c**) Mean inhibitory weight. (**d**) Mean changes in the weight of the inhibitory synaptic afferents. (**e**) Rate of STP change (note that both fixed-STF and STD remain fixed, shown as dashed lines). (**f**) Area between normalised excitatory and inhibitory tuning curves (cf. h-j) during the course of simulated development. A normalised area close to 0 represents a perfectly balanced neuron. (**g**) Additional statistics for the three models. (i) Total neuronal activity calculated using the area between the firing rate in (b) and the desired target rate of 5 Hz. (ii) Average coefficient of variation of the firing rates across simulated development (cf. (b)). (iii) Percent of time spent under homeostasis (i.e. at the desired firing rate; cf. (b)). (iv) Average change in inhibitory weights (cf. (d)). (**h-j**) Snapshots of excitatory and inhibitory tuning curves across three points in simulated development: 10s (star), 1000s (square) and 10 000s (triangle). Shaded gray area represents difference between excitatory and inhibitory tuning curves (cf. (f)). (**h-j**) Excitatory (red) and inhibitory (blue) postsynaptic tuning curve for the fixed-STD (h), fixed-STF (i) and dev-STP models (j).

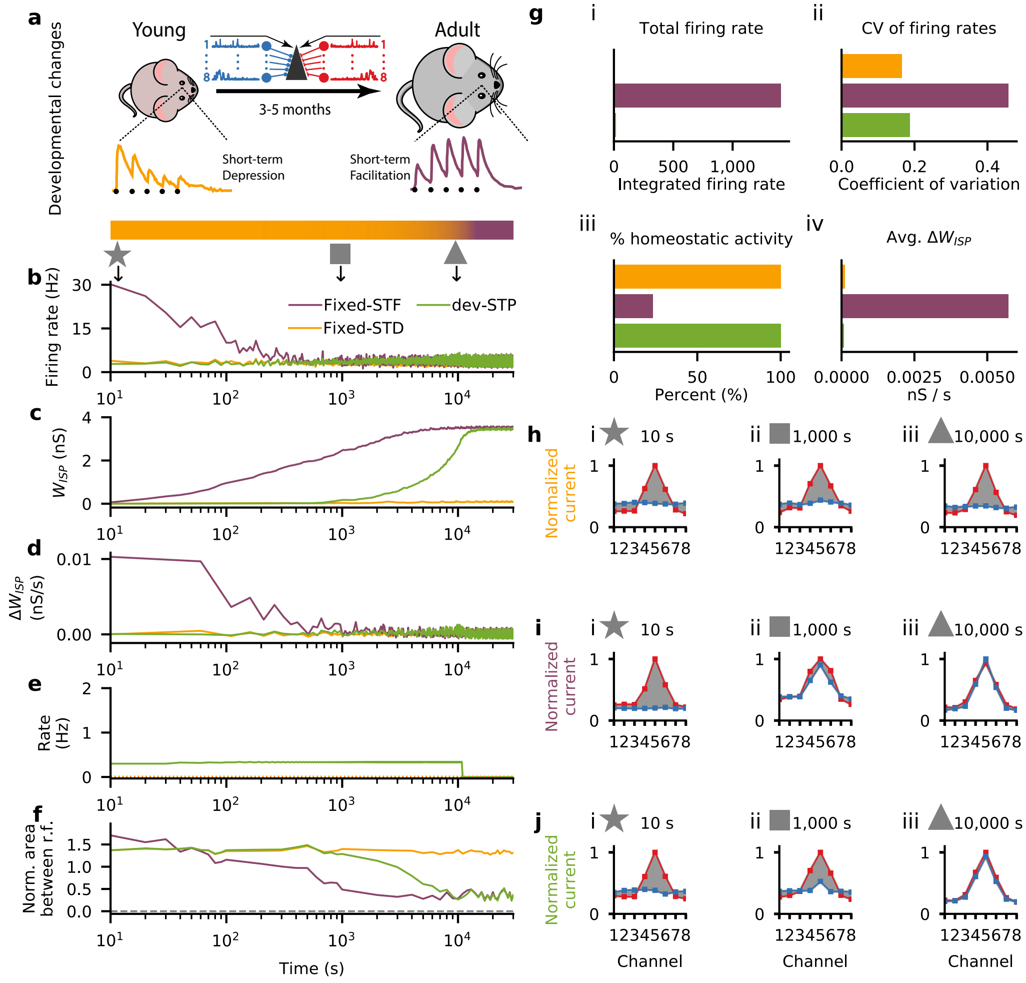

Figure S11. Developmental STP model in which STP changes are pre-defined. (**a**) Schematic of our developmental short-term plasticity (STP) model (cf. Fig. S2); top: young and adult STP (as in Fig. 1); bottom: gradual changes in STP from depressing to facilitating dynamics (orange and purple respectively, in log-scale as in b-f). (**b**-**f**) Different variables of the model across simulated development for three different models: fixed short-term depression (fixed-STD, orange), fixed short-term facilitation (fixed-STF, purple) and developmental model with gradual changes in STP (dev-STP, green line). Note x-axis on log-scale. (**b**) Receiver neuron firing rate. (**c**) Mean inhibitory weight. (**d**) Mean changes in the weight of the inhibitory synaptic afferents. (**e**) Rate of STP change (note that both fixed-STF and STD remain fixed, shown as dashed lines). (**f**) Area between normalised excitatory and inhibitory tuning curves (cf. h-j) during the course of simulated development. A normalised area close to 0 represents a perfectly balanced neuron. (**g**) Additional statistics for the three models. (i) Total neuronal activity calculated using the area between the firing rate in (b) and the desired target rate of 5 Hz. (ii) Average coefficient of variation of the firing rates across simulated development (cf. (b)). (iii) Percent of time spent under homeostasis (i.e. at the desired firing rate; cf. (b)). (iv) Average change in inhibitory weights (cf. (d)). (**h**-**j**) Snapshots of excitatory and inhibitory tuning curves across three points in simulated development: 10s (star), 1000s (square) and 10 000s (triangle). Shaded gray area represents difference between excitatory and inhibitory tuning curves (cf. (f)). (**h**-**j**) Excitatory (red) and inhibitory (blue) postsynaptic tuning curve for the fixed-STD (h), fixed-STF (i) and dev-STP models (j).

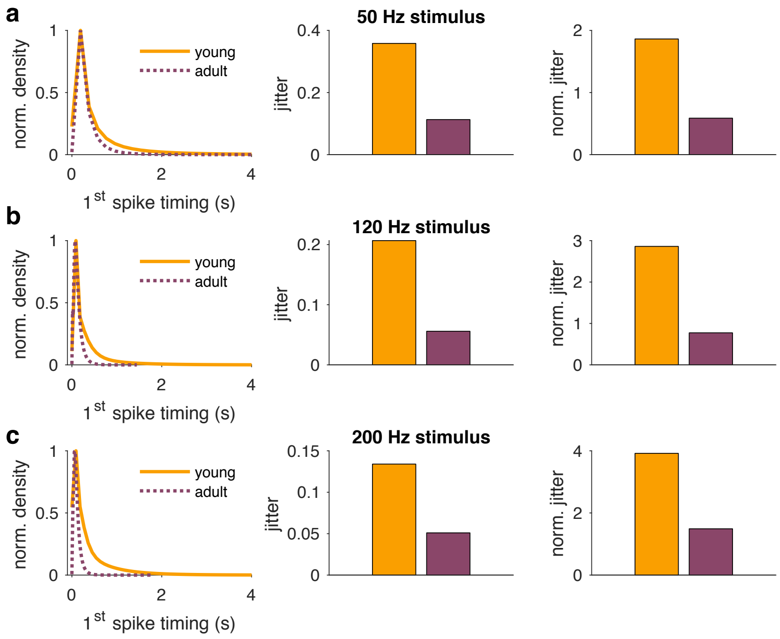

Figure S12. Spike timings and jitter for young and adult conditions in the dev-STP model. (**a**) Density of the first spike timings (left), jitter (i.e. standard deviation of the spike timings, middle) and normalised jitter (right) for a 50 Hz input stimulus. (**b**,**c**) Same as (a) but for a 120 Hz and 200 Hz input stimulus, respectively (cf. Fig. 6).

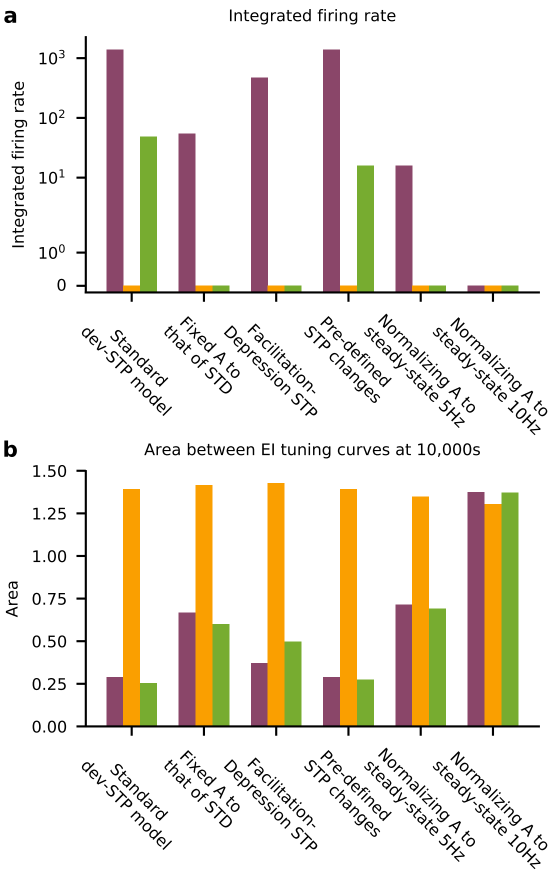

Figure S13. Summary of results for all models. (**a**) Summary plot of the integrated firing rate for each of the models tested, relating to (Fig. 2g.i); note that y-axis is log-scale. (**b**) Area between the EI tuning curves at 10,000 seconds for each of the models tested, relating to (Fig. 2h.iii, i.iii, j.iii). A perfect E-I balanced would have an area of zero.

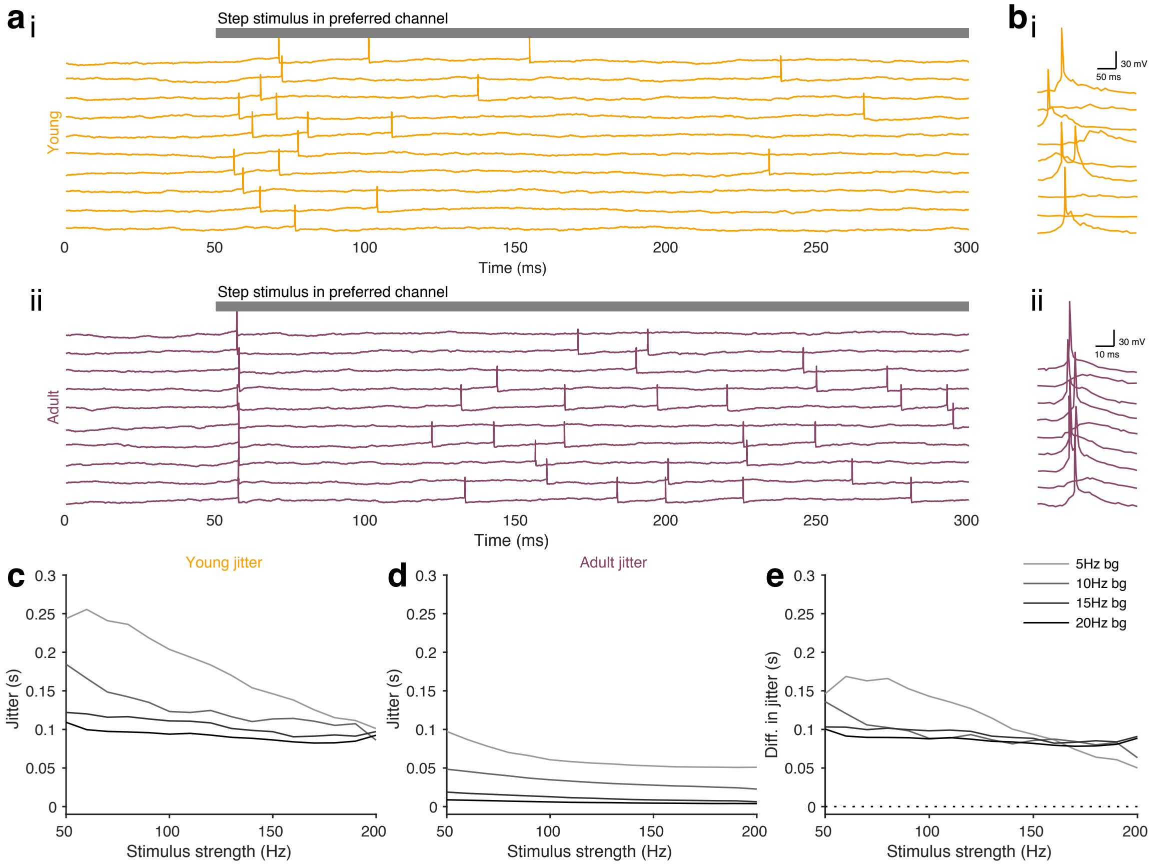

Figure S14. Adult STP improves temporal precision of postsynaptic spikes - non-normalized jitter. (**a**) Examples of postsynaptic voltage responses with preferred-channel input for both young STP model (i) and adult STP model (ii); gray bar at top represents time during which preferred channel is stimulated. (**b**) Stimulus evoked responses in *in vivo* recordings across a few trials in young (i) and adult (ii) animals. Panels adapted from a previous study [26]. In (a,b) the background firing rate is 5 Hz. (**c,d**) Jitter of postsynaptic spikes in the young (c) and adult (d) model for different background firing rates (denoted by different shades of gray; see Methods; cf. Fig. S12). (**e**) Difference between jitter of young STP model (c) and adult STP model (d).

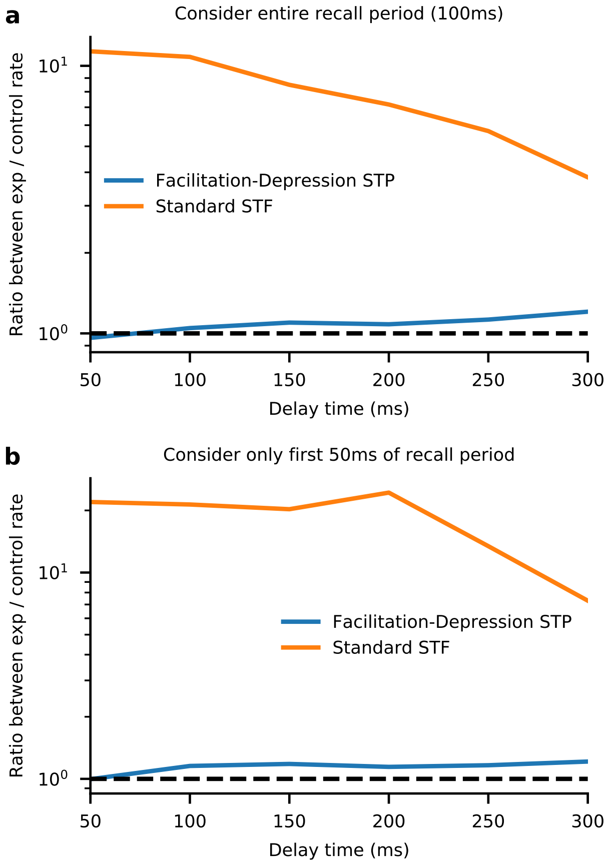

Figure S15. Short-term memory analysis comparing standard STF model and facilitation-depression STP model. We tested a range of delay times (50ms to 300ms) and plot here the ratio of the firing rate during the recall period between the experimental (memory preloaded) protocol and the control protocol. Two models are considered, the standard STF model which sets the excitatory STP to be the STF parameters in Table 2 (orange lines), and the facilitation-depression STP model which sets the excitatory STP to be the parameters in Table 4 (blue lines). (**a**) considers only the average firing rate during the entire recall period of 100ms; (**b**) considers only the firing rate during the first 50ms of the recall period.

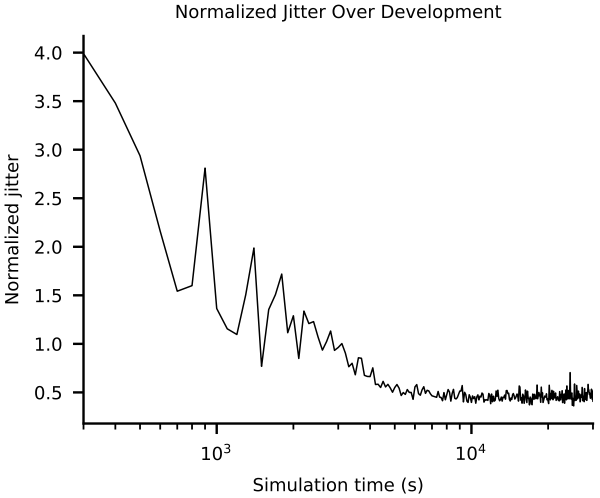

Figure S16. Jitter over development for devSTP model. Normalized jitter over development for the case of 150Hz input.

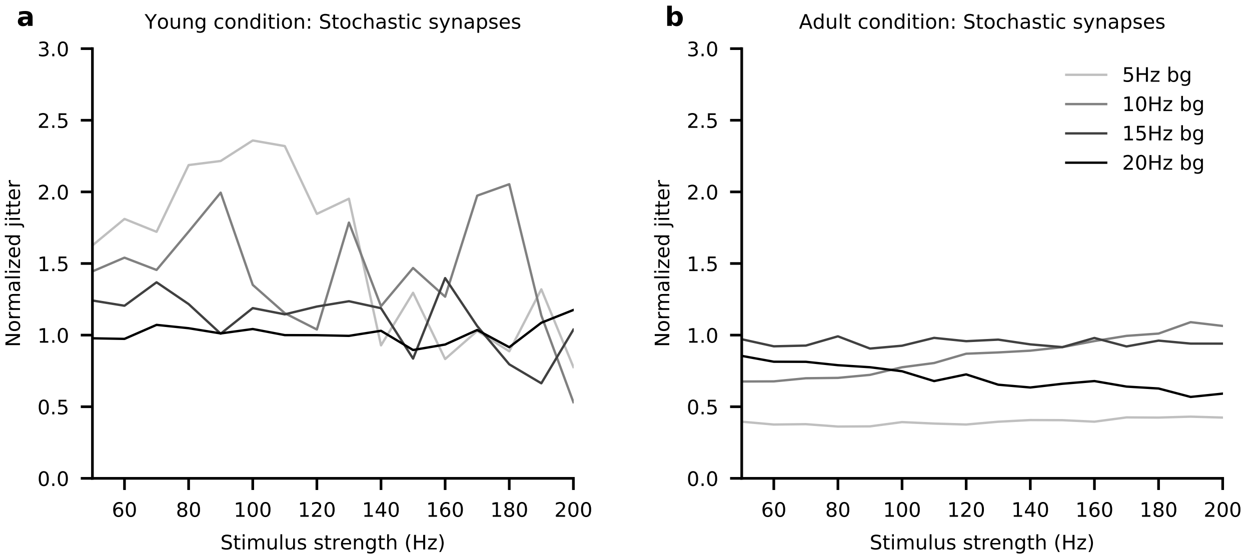

Figure S17. Jitter in devSTP model with stochastic synapses. (**a,b**) Normalized jitter of postsynaptic spikes in the young (a) and adult (b) model for different background firing rates (denoted by different shades of gray.

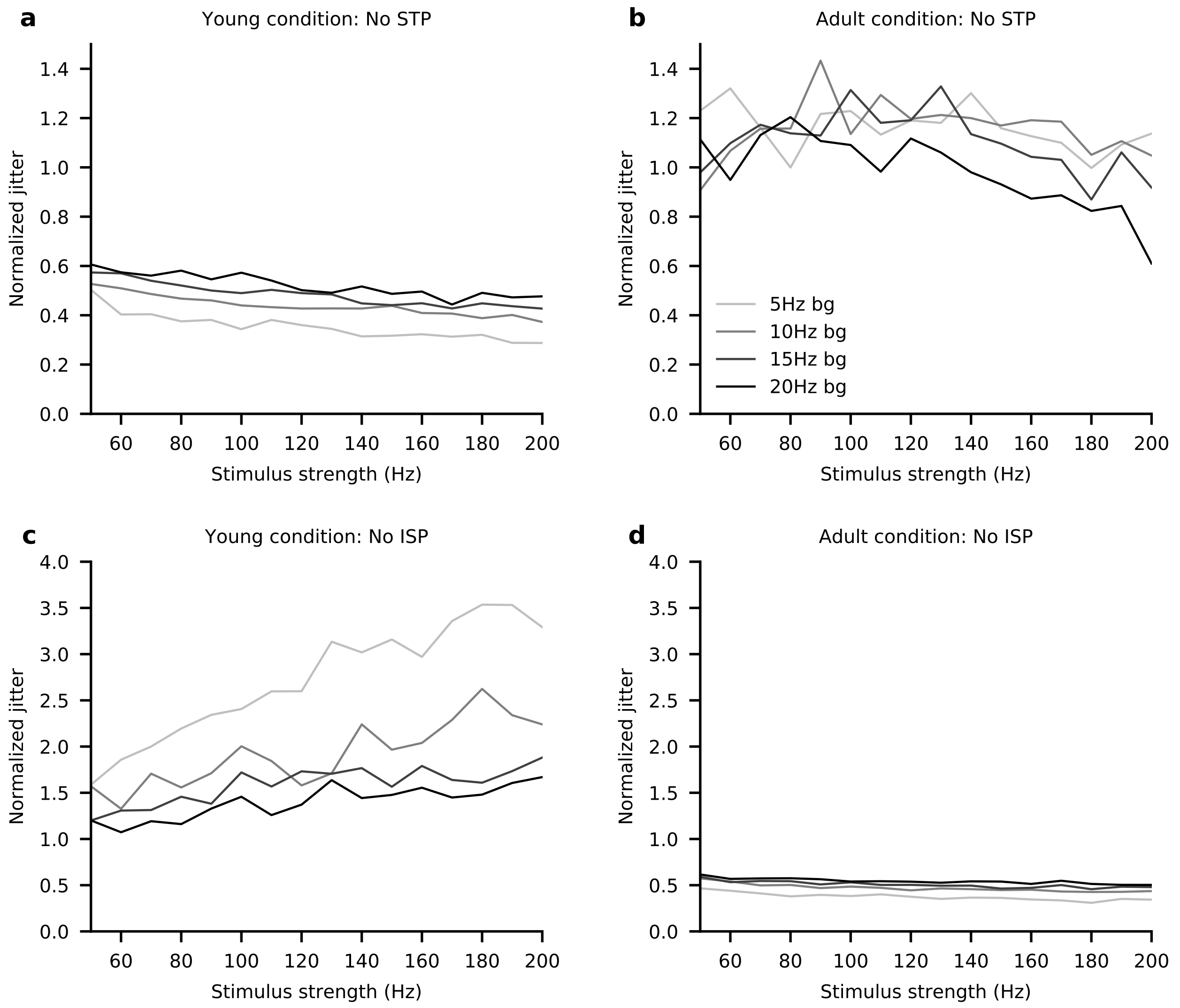

Figure S18. Jitter in models without developmental changes in STP or ISP. (**a**,**b**) Jitter in young (a) and adult (b) conditions in a model without STP. (**c**,**d**) Jitter in young (c) and adult (d) conditions in a model without ISP.

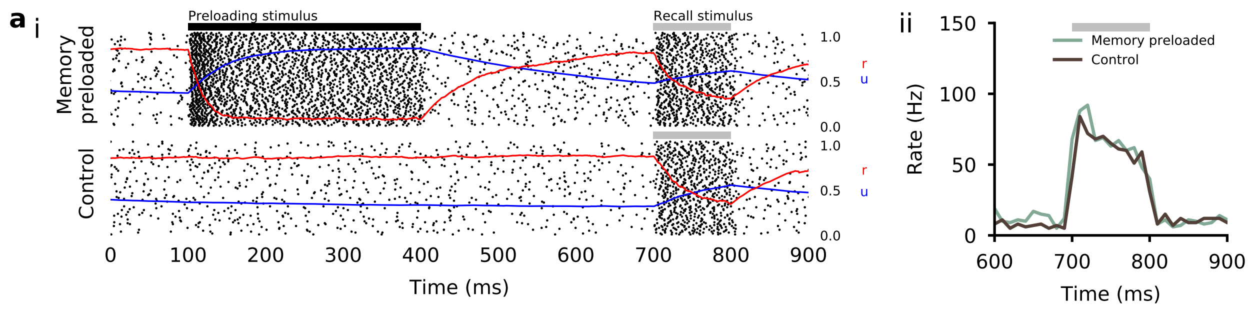

Figure S19. DevSTP model without ISP shows lack of short-term memory traces. (**i**) Raster plot of short-term memory test (SMT; top) with a preloaded stimulus and subsequent recall stimulus (black and gray bars respectively) compared with raster plot of trials without the preloaded stimulus (bottom). Average firing rates (**ii**) for both memory preloaded (light green) and control conditions (dark brown). Release probability (u in blue) and number of presynaptic resources (R in red) are also given for reference (see Methods).
